# Assessment of pathogenic potential in non-pathogenic industrially relevant bacteria

**DOI:** 10.1099/acmi.0.001079.v3

**Published:** 2026-01-30

**Authors:** Katrine Nøhr-Meldgaard, Carsten Struve, Hanne Ingmer, Yvonne Agersø

**Affiliations:** 1 Chr. Hansen A/S, Hørsholm, Denmark. Now Novonesis A/S, Lyngby, Denmark; 2 Department of Veterinary and Animal Sciences, University of Copenhagen, Frederiksberg, Denmark

**Keywords:** lactic acid bacteria, pathogenicity, virulence

## Abstract

Assessment of the pathogenic potential (virulence and toxicity) in non-pathogenic bacterial species is a challenge as it relies on methods developed for assessment of species known to be pathogenic. Here, we have applied and evaluated some of these methods on industrially relevant bacteria to differentiate between ‘true’ virulence factors applying only to pathogens and niche factors being defined as promoting colonization and survival rather than pathogenicity and as being present also in non-pathogenic bacteria. We examined the pathogenicity of 49 strains from 9 industrially relevant bacterial species (*Lactobacillus gasseri*, *Lactobacillus jensenii*, *Lactobacillus delbrueckii*, *Lacticaseibacillus rhamnosus*, *Limosilactobacillus fermentum*, *Latilactobacillus curvatus*, *Ligilactobacillus salivarius*, *Staphylococcus carnosus* and *Staphylococcus xylosus*), including 14 clinical isolates of the same species, through genomic screening and phenotypically through assays established for pathogenic bacteria. The genomes were screened against the Virulence Factor Database (VFDB), and thresholds (>80% nucleotide or protein identity, >70% coverage) provided by the European Food Safety Authority (EFSA) were adopted to differentiate between genes of potential concern and genes of no concern. Core genome analysis was performed to determine whether the clinical isolates were phylogenetically related to the industrial isolates. The genotypic assessment did not reveal the presence of true virulence factors in the examined strains, and in the core genome analysis, the clinical isolates could not be distinguished from the industrial strains. Furthermore, cytotoxicity toward Vero cells, negative impact on Caco-2 cell viability and haemolytic activity on blood agar plates were examined, and none of the tested strains exhibited any activity in these assays. Overall, the results suggest that VFDB screening with the EFSA thresholds can be used to differentiate between true virulence factors and niche factors. Furthermore, the use of phenotypic assays supports the genotypic assessment, albeit expert knowledge is required to interpret the results.

## Data summary

Additional data from this study are posted as a supplemental data file with the online version of the manuscript. The genomic sequences for all the clinical isolates and for type strains sequenced in the study were uploaded to NCBI with the NCBI accession numbers found under repositories. Additional NCBI accession numbers of type strains can be found in Table 1.

NCBI accession numbers: JBNYYQ000000000 *Ligilactobacillus salivarius* Ls1; JBNYYR000000000 *Ligilactobacillus salivarius* LMG9476, JBNYYS000000000 *Ligilactobacillus salivarius* LMG9477, JBNYYT000000000 *Latilactobacillus curvatus* LC8, JBNYYU000000000 *Latilactobacillus curvatus* Lc7, JBNYYV000000000 *Latilactobacillus curvatus* Lc6, JBNYYW000000000 *Latilactobacillus curvatus* Lc5, JBNYYX000000000 *Latilactobacillus curvatus* NCFB2739, JBNYYY000000000 *Limosilactobacillus fermentum* Lf8, JBNYYZ000000000 *Limosilactobacillus fermentum* Lf7, JBNYZA000000000 *Limosilactobacillus fermentum* Lf6, JBNYZB000000000 *Limosilactobacillus fermentum* Lf5, JBNYZC000000000 *Limosilactobacillus fermentum* Lf4, JBNYZD000000000 *Limosilactobacillus fermentum* Lf3, JBNYZE000000000 *Limosilactobacillus fermentum* LMG6902, JBNYZF000000000 *Lactobacillus delbrueckii* subsp. *bulgaricus* Ld1, JBNYZG000000000 *Lactobacillus delbrueckii* subsp. *bulgaricus* ATCC 11842 = JCM 1002 DSM 20081, JBNYZH000000000 *Lactobacillus jensenii* LMG6414, JBNYZI000000000 *Lactobacillus gasseri* Lg3, JBNYZJ000000000 *Lactobacillus gasseri* Lg2, JBNYZK000000000 *Lactobacillus gasseri* ATCC 33323=JCM 1131 DSM 20243.

**Table 1.** Species included in the present study

Species	Type strains	QPS status	Origin
*L. delbrueckii* (4)*	subsp. *bulgaricus* DSM20081 (NCBI accession no. JBNYZG000000000) subsp. *delbrueckii* DSM20074 (NCBI accession no. CP018615) subsp. *lactis* DSM20072 (NCBI accession no. CP022988)	QPS	Blood sample (1)*, Bulgarian yoghurt (1), sour grain mash (1), Emmental cheese (1)
*L. gasseri* (4)	DSM20243 (NCBI accession no. JBNYZK000000000)	QPS	Blood sample (2), human (1), human faeces (1)
*Lactobacillus jensenii* (1)	LMG6414 (NCBI accession no. NZ_AZCQ01000001)	Non-QPS	Human vaginal discharge
*L. rhamnosus* (7)	LMG6400 (NCBI accession no. JBNYZK000000000)	QPS (risk group 2 in Germany, risk group 1 if strains have a long history of safe use)	Healthy human (2), human faeces (2), healthy human intestinal flora (1), Finnish cheese (1), Swiss Emmental cheese (1)
*L. curvatus* (9)	NCFB2739 (NCBI accession no. JBNYYX000000000)	QPS	Blood sample (4), Danish sourdough (1), meat sample (2), milk (1), NA (1)
*L. salivarius* (3)	subsp. *salicinius* LMG9476 (NCBI accession no. JBNYYR000000000) subsp. *salivarius* LMG9477 (NCBI accession no. JBNYYS000000000)	QPS	Blood sample (1), saliva (2)
*L. fermentum* (9)	LMG6902 (NCBI accession no. JBNYZE000000000)	QPS	Ascites fluid (1), blood sample (5), fermented beets (1), human faeces (2)
*S. carnosus* (7)	subsp. *carnosus* DSM20501 (NCBI accession no. NZ_UHCT01000002) subsp. *utilis* DSM11676	Non- QPS	Dry sausage (1), fermented fish sauce (1), meat sample (1), NA (4)
*S. xylosus* (5)	DSM20266 (NCBI accession no. NZ_UHEI01000002	Non-QPS	Stilton cheese (1), human skin (1), Parma ham (1), na (2)

* Number of isolates in parenthesis. na: not available; QPS: qualified presumption of safety [17].

## Introduction

Virulence factors are defined as structures or strategies that contribute to the infectious potential of a pathogenic microbe, which can be categorized into those that promote colonization and survival and those that cause damage to the host [1]. As argued by Hill [1], the genes that promote colonization and survival, e.g. bile tolerance, immune evasion in non-sterile body sites, macro- and micronutrient acquisition, attachment mechanisms and various other colonization and microbe–host communication strategies, are factors often shared by harmless commensal organisms and are involved with survival, growth and nutrient acquisition and should therefore be referred to as niche factors. Hill proposes that ‘*a* “*true*”* virulence factor is a product, structure or strategy that helps a microbe gain access to or survive in normally noncolonized body sites or cellular compartments (e.g., internalins and invasins), cause damage to the body (e.g., cytolytic or hemolytic toxins), cause dysregulation of the immune system to the extent of creating disease symptoms (e.g., superantigens), or cause a neurological response that again leads to disease symptoms (e.g., neurotoxins)*’ [1].

Lactobacilli are present naturally as part of the gut intestinal flora [2]. Several probiotic bacteria (living micro-organisms that confer a health benefit when ingested) were originally isolated from the gastrointestinal tract and are therefore adapted to it and, for that reason, possess colonization and survival strategies known from pathogens [1]. For example, tad type IV pili, which are generally considered virulence factors, are widely distributed in non-pathogenic *Bifidobacterium* species and have been shown to promote colonization and competition ability in the gastrointestinal tract [3]. However, due to the focus on pathogenic bacteria in the scientific literature, adhesion and several other strategies promoting colonization are considered as virulence factors. Virulence factors and niche factors are therefore both included in databases such as the Virulence Factor Database (VFDB). The lack of differentiation between virulence factors and niche factors can create regulatory compliance challenges when the term virulence factor is used to describe gene products and strategies that are widely disseminated in cohabitating commensal microbes. Furthermore, true virulence factors have not been identified in any lactobacilli species [1,4,9], and a study suggests that the greatest safety concern in relation to lactobacilli is if strains horizontally acquire virulence genes from pathogenic microorganisms, which potentially can increase pathogenicity, although no concrete examples have been reported [10].

The incidence of infections in healthy individuals caused by lactobacilli is low. For example, rates of bacteraemia did not increase in Finland when the use of probiotics increased from 1990 to 2000 [11,12]. However, patients with underlying conditions such as compromised immune systems, structural heart disease, cancer patients, pre-term infants and patients undergoing prolonged hospitalization are at risk of developing endocarditis and bacteraemia caused by non-pathogenic bacteria. Therefore, the potential vulnerability of the consumer or patient, dose and duration of consumption should be considered before administering probiotics [8]. Moreover, the frequency of endocarditis caused by lactobacilli is low (0.05–0.4% of total number of bacterial endocarditis) [11,13,16], and in most of the clinical cases, the infections are polymicrobial with the possibility of misidentification of some isolates. Altogether, infections caused by lactobacilli are rare, and most cases are related to predisposed individuals rather than the bacterial strain being virulent. In several cases, the bacteria originated from the patient’s own gut microflora [4,11, 12].

Several bacterial species used for industrial purposes in food and feed are considered safe for use based on a large body of knowledge of the species as evaluated by the European Food Safety Authority (EFSA) and have obtained a qualified presumption of safety (QPS) status [17]. With regard to virulence, the QPS status can be acquired if the species does not possess any safety concerns such as the presence of virulence factors or toxins that may make it pathogenic in humans and animals. If clinical cases exist, it must be evaluated whether it affected patients with underlying diseases or immunosuppression and if transmission occurred through food or other routes, e.g. medical devices [18,19]. Additional qualifiers at the strain level may be relevant, e.g. assessment for cytotoxicity for QPS *Bacillus* species [20]. The QPS list only includes species evaluated by EFSA, and it is, therefore, not comprehensive. For example, *Staphylococcus carnosus* and *Staphylococcus xylosus* are non-QPS species despite being used for industrial purposes since the 1950s [21,22]. Some countries have additional guidelines, e.g. the Committee on Biological Agents (ABAS) in Germany provides a classification of prokaryotes into risk groups, where *Lacticaseibacillus rhamnosus* is included in the risk group 2, with strains used safely for technical applications over many years assigned to risk group 1[23]. Probiotics with a history of safe use in food are considered traditional food ingredients and are legally permitted for human consumption in most jurisdictions (e.g. the USA and the EU) without pre-market authorization [19].

When live bacteria are intentionally included in food and feed products, documentation is required that the strain is unable to cause infection in consumers after ingestion[24]. In the 2018 EFSA guidance, it is a requirement to perform a genome screening using up-to-date databases such as the VFDB [25,26], Pathogenicity Island Database [27,28] or MvirDB [29], unless the species qualify for QPS status [EFSA panel on Additives and Products or Substances used in Animal Feed (FEEDAP), 2018]. Genes and proteins with identity above 80% and coverage above 70% should be reported, and phenotypic assessment may be required [30]. Applying the 80% identity and 70% coverage thresholds reduces the risk of false positives, such as proteins that share a conserved domain with a virulence gene but have a different function. Due to the lack of tools developed for non-pathogenic bacteria, databases such as VFDB are used, even though studies have shown that these tools are not optimal for assessment of non-pathogenic bacteria due to inclusion of fitness factors and lack of interpretation criteria [only recently have the EFSA thresholds been published (80% identity and 70% coverage)] [9]. Furthermore, phenotypic assays for assessing virulence in non-pathogenic bacteria mostly consider cytotoxicity and haemolytic activity [20,31, 32].

Tools to correctly differentiate between true virulence factors and niche factors are therefore needed for non-pathogenic bacteria, as the current tools available to assess virulence have been developed from the available knowledge which is mainly from the clinical area. Furthermore, there is a shortage of standardized phenotypic assays for assessing virulence in non-pathogenic bacteria.

The aim of the present study was to assess the pathogenicity of 9 industrially relevant bacterial species (49 strains in total, including clinical isolates for 5 of the 9 species). The assessment was done to differentiate between true virulence factors and niche factors. Pathogenicity was assessed by screening the genomes against the VFDB and performing phenotypic assays developed originally for pathogenic bacteria. Furthermore, the relatedness between the clinical and industrial isolates was further examined through core genome analysis. These analyses contribute to a better understanding of the challenges with assessment of pathogenicity when genes of potential concern are found in genomic screenings.

## Methods

### Bacterial strains and selection criteria

A total of 49 bacterial strains belonging to nine bacterial species were included in the present study (Table 1). The strain collection included the type strains from each species and representative isolates from Chr. Hansen’s Culture collection, hereafter termed ‘industrial strains’. Fourteen of the 49 bacterial strains which were clinical strains, isolated from blood and ascites fluid, belonging to 5 of the 9 species (*Lactobacillus delbrueckii*, *Lactobacillus gasseri*, *Latilactobacillus curvatus*, *Ligilactobacillus salivarius* and *Limosilactobacillus fermentum*) were included. The clinical isolates were purchased from Statens Serum Institut (SSI), Denmark. All the clinical strains were isolated in the period 2005–2010. No detailed clinical data are available, including patient risk factors or whether the organism contributed to the infection. However, the strains were isolated from blood and ascites fluid exclusively from individuals from 65 to 86 years of age for women and 52–83 years of age for men. All strains were stored in the Chr. Hansen’s Culture collection at −80 °C.

The industrially relevant species were selected to represent a broad spectrum of properties. *S. carnosus* and *S. xylosus* were included since they belong to the *Staphylococcus* genus which includes pathogenic bacteria, and virulence factors from this genus are included in the VFDB. *L. rhamnosus* was included since it is found more frequently in relation to human infection than other lactic acid species, and *L. rhamnosus* strains that have not previously been used for technical applications are categorized in the risk group 2 by the Committee on Biological Agents (ABAS) in Germany [13; Committee on Biological Agents (ABAS), 2010]. *L. jensenii* was included as it is not included in the QPS list from EFSA, since it has not been evaluated by EFSA. The five QPS species *L. delbrueckii*, *L. gasseri*, *L. curvatus*, *L. salivarius *and *L. fermentum* for which clinical isolates were available were included to examine potential differences between clinical and industrially relevant non-clinical strains.

### Growth condition used prior to experiments

Prior to experimental assays, all strains were grown anaerobically using AnaeroGen™ Sachet (Oxoid) in De Man, Rogosa, Sharpe (MRS) medium (Oxoid) [33], except strains of *S. carnosus* and *S. xylosus*, which were grown aerobically in brain heart infusion (BHI) medium (Oxoid). *L. fermentum*, *L. curvatus*, *S. xylosus* and *S. carnosus* were incubated at 30 °C, while the remaining five species were incubated at 37 °C. The pathogenic control *Staphylococcus aureus* JE2 strain (accession number CP020619) was grown aerobically in Tryptic Soy Broth (TSB) at 37 °C, and *Streptococcus equi* subsp. *zooepidemicus* strain CCUG23256 (kindly provided by Professor Anders Miki Bojesen, Department of Veterinary and Animal Sciences, University of Copenhagen) was grown aerobically in BHI at 37 °C. Growth conditions for specific assays are described under the relevant section.

### Genomic DNA extractions, library preparation and QC for *de novo* short-read (Illumina) whole-genome sequencing

Genomic DNA for *de novo* short-read whole-genome sequencing was extracted from bacterial cell pellets harvested from 1 ml of overnight culture normalized to OD_600_= 1. Clean Blood and Tissue DNA Kit (NACBT-D0384) (Clean NA, The Netherlands) was used, and the manufacturer’s protocol was modified. The extraction method was automated and performed on Biomek i5 Liquid Handler (Beckman Coulter, USA). Modifications to the manufacturer’s protocol: cell pellets were resuspended in 200 µl of pre-lysis buffer (PBS, 20 mg ml^−1^ lysozyme, 50 U/mutanolysin, 100 mg ml^−1^ RNase A) instead of the Tissue Lysis buffer supplied in the kit [34].

Genomic libraries were generated for most of the strains using modified Kapa HyperPlus Library Preparation Kit (Roche, Switzerland) on Biomek i5 Liquid Handler (Beckman Coulter, USA). A quantity of 150 ng of genomic DNA diluted in 15 µl EB buffer (Tris-Cl, pH 8.0) was used in the half-volume reaction mixes for fragmentation, end-repair/A-tailing, ligation and final amplification. A total of 0.1 mM conditioning solution was added to fragmentation mix, and fragmentation time was optimized to 10 min. Five microlitres of 1 µM Kapa Dual-Indexed adapter (Roche, Switzerland) were used during the adapter ligation step. Ten microlitres of the adapter-modified DNA fragments were enriched by 8-cycle PCR. Clean NGS beads (Clean NA, The Netherlands) were used for two post-ligation and two post-amplification clean-ups to purify fragments at an average size between 450 and 550 bp [34].

For 15 of the strains, genomic libraries were generated using NEBNext^®^ Ultra^™^ II FS DNA Library Prep Kit for Illumina^®^ with NEBNext Multiplex Oligos for Illumina (Unique Dual Index UMI Adaptors DNA Set 1) (New England Biolabs Inc., USA) on Biomek i5 Liquid Handler (Beckman Coulter, USA). Two nanograms of genomic DNA diluted in 15 µl EB buffer (Tris-Cl, pH 8.0) were used in the half-volume reaction mixes for fragmentation, end-repair/A-tailing, ligation and final amplification. Fragmentation time was optimized to 8 min. Five microlitres of 2.5 µM NEBNext Multiplex Oligos for Illumina (Unique Dual Index UMI Adaptors DNA Set 1) (New England Biolabs Inc., USA) were used during adapter ligation step. Ten microlitres of the adapter-modified DNA fragments were enriched by 9-cycle PCR. Clean NGS beads (Clean NA, The Netherlands) were used for double-sided post-ligation size selection and one post-amplification clean-up to purify fragments at an average size between 450 and 550 bp.

The concentration of genomic DNA and dsDNA libraries was measured by Qubit Flex^®^ Fluorometer using Qubit dsDNA Broad Range and Qubit 1x dsDNA HS assays (Thermo Fisher Scientific, USA), respectively. Average dsDNA library size distribution was determined using the Agilent HS NGS Fragment (1–6,000 bp) kit on the Agilent Fragment Analyzer (Agilent Technologies, Santa Clara, USA). Libraries were normalized and pooled in the normalization buffer (10 mM Tris-Cl, pH 8.0, 0.05% Tween 20) to the final concentration of 10 nM [34].

Denaturized in 0.2 N NaOH, 1 pM pool of libraries in 13,00 µl ice-cold HT1 buffer was loaded onto the flow cell provided in the NextSeq Reagent Mid Output (300 cycles) and sequenced on a NextSeq platform (Illumina, USA) with a paired-end protocol and read lengths of 151 nt [34].

### Genome assembly

All processing of the short reads was done in either CLC Genomics Server version 20.0.5 or CLC Genomics Workbench version 20.0.5.

The short reads were mapped with default parameters to the reference sequence of the phage Phi X 174 using the tool ‘Map reads to reference’. Unmapped reads from the mapping were trimmed for quality using the PHRED score 23 as the threshold and with the non-default parameter of discarding reads that were less than 50 bp long using the tool ‘Trim Sequences’ [34].

The trimmed reads were *de novo* assembled with default parameters except for the minimum contig length which was set to 350 bp using the tool ‘*De Novo* Assembly’. Afterwards, a decontamination step was performed where contigs with low depth of coverage were removed using a custom plugin written by Qiagen. The decontamination step first removes all contigs where the depth of coverage is below 15X and afterwards removes all contigs where the depth of coverage is below 25% of the median depth of coverage for the entire genome assembly [34].

Gene calling of the filtered contigs was done with Prodigal version 2.6.3 using the default parameters.

### Species identification, core genome analysis and VFDB screening

For each species, where more than one strain was included, core genome analysis was performed to enable species identification, ensure that the strains were not identical and examine whether the industrially used strains and the clinical isolates belong to phylogenetically different subgroups. In brief, the genomes, either fully assembled or contigs, were annotated by the software tool Prokka, which annotates genomes using different tools including Prodigal (CDSs), RNAmmer (rRNA genes), Aragorn (transfer RNA genes), SignalP (signal leader peptides) and Infernal (non-coding RNA) [35]. Prokka annotation is a requirement for using Roary, since the .gff file (file containing sequences and annotations) provided by Prokka is used by Roary to create a multi-FASTA alignment of all the core genes [36]. Roary was set to perform nucleotide alignment using MAFFT and a blastp percentage identity between 80 and 100%, depending on species [37]. FastTree was used to produce an approximately maximum-likelihood phylogenetic tree from the core gene alignment file, which was visualized by mega X [38,40].

The VFDB core dataset was downloaded in nucleotide and protein level formats (http://www.mgc.ac.cn/VFs/download.htm) and imported to CLC Genomics Workbench 20.0.5 (Qiagen Bioinformatics, Aarhus, Denmark) on 20 October 2020. The assembled contigs of each strain were joined using the join function in CLC and annotated using the Rapid Annotation using Subsystems Technology (RAST) server with default settings [41,42]. The annotated genomes were subjected to BLAST analysis against the VFDB in CLC with the following settings: word size 3, E-value cut-off 1E-50 for BLASTx and word size 11 and E-value cut-off 1E-10 for BLASTn. Thresholds (80% identity and 70% coverage at nucleotide and protein level) were adopted from EFSA [30].

### Assessment of haemolytic activity

To assess haemolytic activity, the strains were streaked on tryptone soya agar plates with sheep blood (Thermo Fisher Diagnostics ApS) and incubated under aerobic conditions for 24 h. *S. aureus* JE2 strain (accession number CP020619) was included as a pathogenic positive control.

### Hydrogen peroxide production

All strains were streaked on agar plates (lactobacilli on MRS agar plates, staphylococci on BHI agar plates) supplemented with azino-bis-3-ethylbenzothiazoline-6-sulphonic acid (ABTS) (1 mg/ml per 1 l of MRS) (Sigma-Aldrich) and HRP (250 mg HRP/litre MRS) (Sigma-Aldrich). Plates were incubated for 24 h under anaerobic conditions. After the end of incubation, the plates were exposed to air for 30 min, followed by visual inspection of green colour development. Greenish pigment is formed when the HRP generates oxygen from hydrogen peroxide (H_2_O_2_) produced by the strains, which in turn oxidizes the ABTS substrate [43,44]. H_2_O_2_ production according to the intensity of green colour development was scored: strong production (+++) for dark green, intermediate production (++) for green, weak production (+) for light green and non-producer (-) for no green colouration [43,45].

### Vero cell cytotoxicity assay

For the Vero cell cytotoxicity assay, culture supernatants were tested. Culture supernatants were prepared as described by EFSA [24] with some modifications. In brief, 10 ml broth medium was inoculated with cell material from agar plates. Lactobacilli were grown anaerobically, and staphylococci were grown aerobically. The following day, 100 µl overnight culture was transferred to 20 ml prewarmed broth medium and incubated. For sampling, 8 ml of broth was collected, centrifuged at 5,900 r.p.m. (~3,814 ***g***) for 10 min, and the supernatant was sterile-filtered to obtain cell-free samples and stored at −80 °C.

Samples were collected after 24 and 48 h of incubation for all the included species. *S. carnosus* and *S. xylosus* samples were furthermore collected after 6 h of incubation. As a negative control, 2 ml of sterile broth medium was collected and stored at −80 °C, and a 6 h sample of *Bacillus cereus* ATCC 14579 was included as a positive control. The purity of the cultures was assessed by streaking on blood agar plates. Detection of cytotoxicity against Vero cells was assessed using the LDH assay performed commercially by Bioneer A/S, Hørsholm, Denmark, according to the recommendation from EFSA [20]. The assay is a colourimetric assay for quantification of cell death and lysis, which measures the activity of lactate dehydrogenase (LDH) released when cells are damaged [46].

### MTT assays

Caco-2 cells were grown in Dulbecco’s Modified Eagle Medium (D-MEM) (Gibco™) with the addition of 1% non-essential amino acids (Gibco™), 20% FBS (Sigma-Aldrich) and 25 µl ml^−1^ gentamycin (Gibco™) at 37 °C with 5% CO_2_.

The MTT [3-(4,5-dimethylthiazol-2-yl)-2,5-diphenyl tetrazolium bromide] assay is a colourimetric assay used to assess cell metabolic activity, where MTT is reduced to the purple formazan crystals by metabolically active cells that contain NAD(P)H-dependent oxidoreductase enzymes [47].

The MTT assay was performed as previously described, although slightly modified [48,49]. In short, 3×10^4^ Caco-2 cells/well were distributed in 96-well plates and incubated for 24 h at 37 °C with 5% CO_2_. After incubation, the cells were seeded, and the clear medium was removed from each well, and 50 µl of D-MEM was added to each well together with 50 µl of bacterial cells diluted in D-MEM+10% FBS. Two concentrations of bacterial cells were used: 1.5×10^7^ and 1.5×10^6^. The plates were incubated for 24 h at 37 °C, after which the medium was removed and 100 µl of 0.5 mg ml^−1^ MTT (Sigma-Aldrich) was added and incubated for a further 2 h at 37 °C. The MTT was removed, and 100 µl DMSO was added to each well and incubated for 15 min with 150 r.p.m. shaking. The absorbance was measured at 570 nm using a BioTek PowerWave™ Microplate spectrophotometer. D-MEM mixed with D-MEM+10% FBS was used as a control, and experiments were conducted in technical triplicates. The Caco-2 cell viability was calculated as follows: % Cell viability=[(ODsample×100)/ODcontrol]. An *S. equi* subsp. *zooepidemicus* strain CCUG23256 was included as a pathogenic control, and Triton X-100-treated cells were also included.

### Hyaluronidase activity

The hyaluronidase activity was examined through an agar plate assay as previously described [50,51]. Agar plates (MRS for lactobacilli, BHI for *S. carnosus* and *S. xylosus* and TSB for *S. aureus*) were prepared with 1 g l^−1^ hyaluronic acid sodium salt (Sigma-Aldrich) and 10 g l^−1^ BSA (Sigma-Aldrich). Plate cultures were flooded with 2 M acetic acid solution for 10 min and subsequently inspected for transparent zones surrounding the colonies. The transparent zones appear due to degradation of hyaluronic acid. The undegraded hyaluronic acid is conjugated with BSA that forms a precipitate that makes the plates appear white [50]. As a positive control, *S. aureus* JE2 wild-type was included, and an *S. aureus hys*A transposon mutant, from the NARSA library, was included as a negative control.

## Results and discussion

### Genotypic assessment of virulence factors by use of VFDB

VFDB was used to screen the 49 included bacterial strains belonging to *L. gasseri*, *L. jensenii*, *L. delbrueckii*, *L. rhamnosus*, * L. fermentum*, *L. curvatus*, *L. salivarius*, *S. carnosus* and *S. xylosus* for the presence of virulence factors. See Tables S1 and S2 (available in the online Supplementary Material) for a VFDB screen at the amino acid level for a non-QPS and QPS strain, respectively.

The VFDB screening only found one gene with identity and coverage above the EFSA threshold (80% identity and 70% coverage at nucleotide and protein level), which was present in the *S. xylosus* Sx3 strain. The gene was annotated as a 6 kDa early secretory antigenic target ESAT-6 (*esx*A).

For the rest of the VFDB-identified genes, identity and coverage were below the EFSA thresholds (80% identity and 70% coverage at nucleotide and protein level), and the proteins were found to be encoded by all or a vast majority of genomes of the same species in the NR NCBI database. This suggests that the genes are conserved within the species [52]. Furthermore, the VFDB search did not reveal the presence of any genes that are considered harmful, e.g. cytotoxic and haemolytic toxins, internalins, invasions, superantigens or neurotoxins, in accordance with previous findings [1,5, 6, 9, 53, 54]. Rather, the genes were found to be involved with survival, growth, attachment and nutrient acquisition in the gastrointestinal tract and should therefore be considered niche factors rather than virulence factors [1].

Besides the VFDB screening, core genome analysis was also performed to examine phylogenetically relatedness of the clinical isolates and the industrial strains. The core genome analysis found no general phylogenetic difference between the clinical isolates and the industrial strains, supporting that the clinical isolates do not belong to a phylogenetically distinct and more virulent subgroup of the species.

**Fig. 1.**
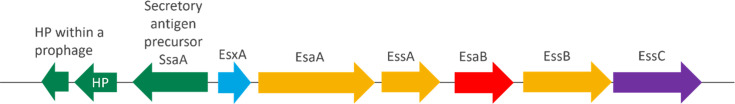
Schematic presentation of the *S. xylosus* strain Sx3 genomic region encoding part of the ESS system from *S. aureus*. Membrane-associated proteins are orange, the EsxA substrate is blue, the EsaB cytoplasmic protein is red, and the FtsK/SpoIII ATPase is purple. Sequences not part of the ESS system are green. HP is hypothetical protein.

The gene encoded by the *S. xylosus* Sx3 strain was annotated as a 6 kDa early secretory antigenic target ESAT-6 (*esx*A) and exhibited 94.8% identity and 100% coverage to a type VII secretion system secreted protein EsxA from *S. aureus* MW2 strain. The *esx*A gene is part of the type VII secretion system ESS, which is required for virulence in murine abscesses, pneumonia and skin-infection models and is highly up-regulated during chronic infections in cystic fibrosis patients [55,58]. Other gene regulatory components (membrane proteins EsaA, EssA, EssB, EssC and soluble protein EsaB) part of the type VII secretion system are also encoded by *S. xylosus* Sx3 (56–71% identity to the corresponding genes in *S. aureus* MW2 strains) (Fig. 1) [59]. This suggests that the gene might confer other functions than in *S. aureus*. Upstream of the EsxA protein, a gene annotated as ‘hypothetical protein within a prophage’ with 75% nucleotide identity and 95% coverage to a gene found in several *S. aureus* genes, is present (Fig. 1), suggesting that the genes might have been acquired. However, the gene was not flanked by any functional mobile genetic elements in proximity.

A BLASTn search reveals that the 6 ESS genes encoded by the *S. xylosus* Sx3 strain, including the hypothetical protein within a prophage, are only found in 1 (accession number NZ_CP066721.1, strain 2.1523, isolated from raw fermented sausages) out of 83 publicly available *S. xylosus* genomes to which the genes exhibit 100% identity and 100% coverage and are positioned in the same genomic position. The genes are therefore not conserved within the *S. xylosus* species. Homologous genes are also found in the non-pathogenic coagulase-negative *Staphylococcus edaphicus* CCM 8731 strain (accession number CP093217) (80% nucleotide identity and 90% coverage), which has been isolated from stone fragments and sandy soil in Antarctica and is one of two publicly available genomes of the species. The genes were also found in five *Staphylococcus caprae* strains, a coagulase-negative skin commensal that can be an opportunistic pathogen with 74.48% identity and 93% coverage (accession numbers CP051643, CP031271, AP018587, AP018586 and AP018585) [60], and several *S. aureus* strains with 72% identity and 99% coverage.

The ESS system is conserved in several Gram-positive bacteria, both pathogenic and environmental bacteria, suggesting that it plays an important role in bacteria not just in relation to virulence [59]. Furthermore, due to the fact that the *S. xylosus* strain only encodes 6 out of the 12 proteins in the *S. aureus* type VII secretion system ESS and lacks the major components conferring virulence (*esx*C, *esx*B, *esx*D and *esa*D), e.g. *esa*D is a nuclease that kills competitor bacteria [59], the functionality and putative role in *S. xylosus* is questionable but most likely not related to virulence. Rather, the system is probably related to the secretion and uptake of substrates; however, the exact function of the ESS system in *S. xylosus* needs to be further clarified.

This illustrates that when databases such as VFDB are used for assessing virulence in non-pathogenic bacteria and the EFSA threshold is applied, expert knowledge is still needed to ensure correct interpretation of the results.

### Phenotypic assessment

#### Assessment of cytotoxicity and Caco-2 viability

Assessment of culture supernatant cytotoxicity using *in vitro* cell-based methods is recommended by EFSA for *Bacillus* species [20], and in the present study, assessment of cytotoxicity using the LDH assay was performed for all the species included. None of the strains exhibited cytotoxicity under the tested conditions, except the positive cytotoxicity control *B. cereus* ATCC14579, in accordance with no cytotoxic or haemolytic toxins being found in the genomic assessment of the nine included species.

The effect of the bacterial cells on the viability of Caco-2 cells was assessed by employing the colourimetric MTT assay, where metabolically active Caco-2 cells can reduce MTT to the purple formazan crystals, thereby differentiating between living and dead Caco-2 cells [48,49].

None of the strains negatively impacted the viability of Caco-2 after exposure, except the pathogenic control *S. equi* subsp. *zooepidemicus*, which was expected as it encodes a streptolysin (Fig. S1) [61].

Interestingly, the reduction of MTT to formazan was high compared to the control when *S. carnosus* and *S. xylosus*, respectively, were added to the Caco-2 cells. Further exploration showed that *S. carnosus* and *S. xylosus* reduce MTT to formazan, and the high absorbance was not caused by an increase in Caco-2 cell viability. The ability to reduce substrates is one of the main reasons why *S. carnosus* strains are used in meat starter cultures, as nitrate or nitrite is added as a curing agent during fermentation but needs to be decreased below a certain threshold level at the end of fermentation [21,62]. The MTT assay is therefore not recommended for assessing human cell viability after exposure to species that are able to reduce substrates.

#### Assessment of haemolytic activity and H_2_O_2_ production

Several Gram-negative pathogenic bacteria produce haemolysins, and assessment of haemolytic activity is therefore commonly used to assess virulence in strains used for industrial purposes [31]. Haemolysins are extracellular and most cause lysis of erythrocytes by forming pores of varying diameters in the membrane, and many also attack other mammalian cells [63]. None of the examined strains, including the clinical blood isolates, exhibited haemolytic activity, except the included pathogenic * S. aureus* JE2 control strain. The lack of haemolytic activity correlated with the genomic assessment where no haemolytic toxins were identified and suggests that lactobacilli isolated from blood have not acquired haemolytic toxins.

All species had strains that produced green colouration of varying intensity on blood agar plates except for *L. fermentum* (Table 2). Green colouration of blood cells results from the oxidation of haemoglobin to methemoglobin in the medium surrounding the colony, causing a greenish/brownish colouration, which is often referred to as α-haemolysis. Microscopic inspection of the greenish/brownish-coloured red blood cells has shown that the cell membrane is intact [64], suggesting that α-haemolysis is a misleading designation as true lysis is not occurring and haemolysin encoding genes are not involved.

**Table 2.** Assessment of ability to cause green colouration of blood and/or produce H_2_O_2_

Species	Subspecies (if relevant)	Strain name	Green colouration of blood in the presence of O_2_	H_2_O_2_ production
*L. gasseri*		DSM20243	+	+
		Lg1	+	+
		**Lg2**	−	−
		**Lg3**	−	−
*L. jensenii*		LMG6414	+	++
*L. delbrueckii*	*bulgaricus*	DSM20081	−	−
	*delbrueckii*	DSM20074	(+)	+
	*lactis*	DSM20072	−	+
	*bulgaricus*	**Ld1**	+	++
*L. rhamnosus*		LMG6400	(+)	−
		Lr1	−	−
		Lr2	−	−
		Lr3	+	−
		Lr4	+	−
		Lr5	(+)	−
		Lr6	(+)	+
*L. fermentum*		LMG6902	−	−
		Lf1	−	−
		Lf2	−	−
		**Lf3**	−	−
		**Lf4**	−	−
		**Lf5**	−	−
		**Lf6**	−	−
		**Lf7**	−	−
		**Lf8**	−	−
*L. curvatus*		NCFB2739	−	−
		Lc1	(+)	−
		Lc2	−	−
		Lc3	(+)	−
		Lc4	−	−
		**Lc5**	−	−
		**Lc6**	+	−
		**Lc7**	+	−
		**Lc8**	−	−
*L. salivarius*	*salivarius*	LMG9477	+	−
	*salicinius*	LMG9476	+	−
		**Ls1**	+	−
*S. carnosus*	*carnosus*	DSM20501	+	−
	*utilis*	DSM11676	+	−
		Sc1	+	−
		Sc2	−	−
		Sc3	+	−
		Sc4	+	−
		Sc5	+	−
*S. xylosus*		DSM20266	−	−
		Sx1	−	−
		Sx2	−	−
		Sx3	(+)	−
		Sx4	(+)	−

+, positive reaction; (+), weak positive reaction; −, no production. Bold strain names represent clinical isolates.

**Fig. 2.**
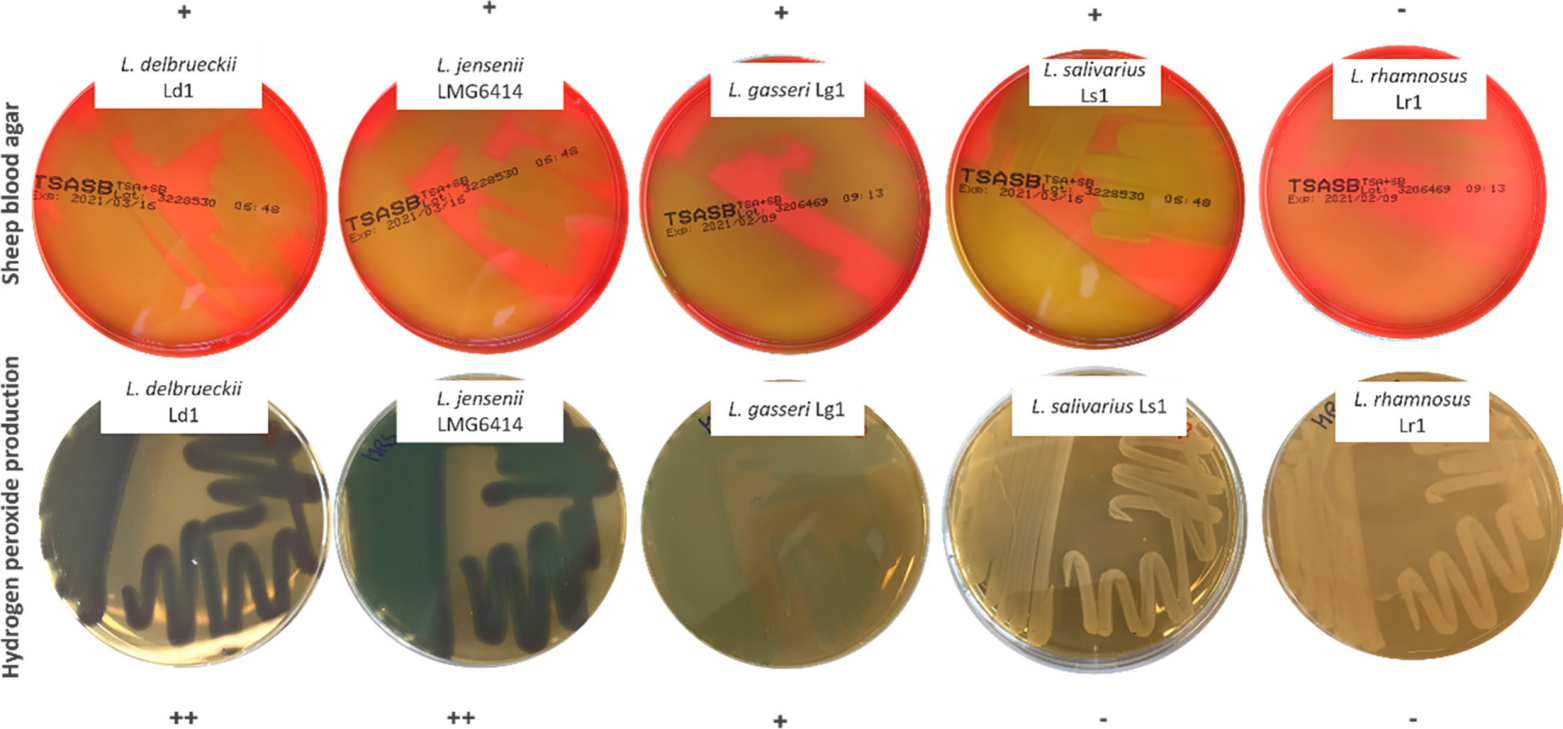
Correlation between green colouration on blood agar plates (top row) and production of H_2_O_2_ (bottom row) for *L. delbrueckii*, *L. jensenii*, *L. gasseri*, *L. salivarius* and *L. rhamnosus.* + and − indicate whether green colouration on blood agar plates or H_2_O_2_ production occurred. *L. salivarius* is included to show that green colouration on blood agar plates can occur without the production of H_2_O_2_.

In different *Streptococcus* species, α-haemolysis is due to the production of H_2_O_2_ [64,68]. In the present study, some of the examined *L. gasseri*, *L. delbrueckii* and *L. jensenii* strains both produced green colouration of blood and produced H_2_O_2_ (Table 2, Fig. 2), which previously has been shown for species found as part of the healthy vaginal microbiota [69].

H_2_O_2_ accumulates in species lacking H_2_O_2_-scavenging enzymes and species adapted to anaerobic environments [69]. H_2_O_2_ is generally believed to be produced via the central carbon and energy metabolisms by oxidases (pyruvate oxidase, lactate oxidase and NADH oxidase) [70] and to be a by-product of metabolism by the enzymes pyruvate oxidase (SpxB) and LDH (LctO) in *Streptococcus pneumoniae* [65]. However, a study has shown that a *Lactobacillus johnsonii* strain produces H_2_O_2_ independently of these oxidases and instead relies on a NADH-dependent flavin reductase, which is also encoded by *L. gasseri*, *L. delbrueckii* and *L. jensenii* [69].

H_2_O_2_ production was previously proposed to be a probiotic property mediated by members of the commensal vaginal microbiota to compete against bacteria associated with bacterial vaginosis. However, this was later shown to be due to the production of lactic acid, which inactivates the bacterial vaginosis-associated bacteria without affecting the vaginal lactobacilli, while the vaginal conditions are not optimal for H_2_O_2_ production [69,71, 72]. Another study has shown that H_2_O_2_ produced by *Lactobacillus crispatus* causes a transient but nontoxic rise in the intracellular reactive oxygen species, affecting the transcriptional activity of the peroxisome proliferator-activated receptor-γ, which is a key regulator involved with preserving mucosal homeostasis by interfering with the activity of proinflammatory transcription factors. This, in turn, was able to significantly reduce the severity of colitis in mice [73], suggesting that H_2_O_2_ production by lactic acid bacteria reduces inflammation and can be characterized as a probiotic factor [1], suggesting that testing for H_2_O_2_ production ability is not a relevant assay for safety assessment of industrial strains within these species.

The remaining five species (*L. rhamnosus*, *L. curvatus*, *L. salivarius*, *S. carnosus* and *S. xylosus*) did not produce H_2_O_2_, despite the ability of some of the strains to produce green colouration of blood. Furthermore, one *L. delbrueckii* subsp. *lactis* strain was shown to produce H_2_O_2_ but did not produce green colouration of blood. Further studies need to be performed to determine the mechanism behind this.

Altogether, there are no indications that α-haemolysis in species considered non-pathogenic is a safety concern.

#### Lack of hyaluronidase activity in *L. rhamnosus*

Hyaluronidases can break down hyaluronic acid and are produced by several pathogenic Gram-positive bacteria that are able to initiate infections at the skin or mucosal surfaces [74]. Group A *Streptococcus* hyaluronidase can digest tissue hyaluronic acid and facilitate the spread of large molecules such as toxins but is not sufficient to cause subcutaneous diffusion of bacteria or to affect lesion size [75].

An ORF annotated as a hyaluronate lyase precursor was found in five out of the six *L. rhamnosus* strains and exhibited 2% protein identity and 74% coverage to a hyaluronidase protein from *Enterococcus faecalis* (accession number EF3023), which is lower than the EFSA thresholds (>80% nucleotide or protein identity, >70% coverage). This gene was accessed further since the gene annotation suggested a relationship with virulence.

Since an assay exists for the assessment of hyaluronidase activity, we examined whether the strains encoding the hyaluronate lyase precursor could break down hyaluronic acid and thereby test whether the EFSA thresholds are useful to predict the genes of potential safety concern. The ability to degrade hyaluronic acid was tested as previously described [50] and included the hyaluronidase producer, *S. aureus* JE2, as well as the *S. aureus* transposon mutant, where the *hylA* gene has been inactivated. None of the strains, except the *S. aureus* wild-type, were able to degrade hyaluronic acid under the examined conditions. A study has previously shown that the *L. rhamnosus* type strain is not able to degrade hyaluronan despite the presence of the hyaluronate lyase precursor, as also shown in this study [76].

Further analysis of the genomic region surrounding the hyaluronate lyase precursor in the *L. rhamnosus* genomes revealed the presence of genes related to carbohydrate metabolism, which, in *Lacticaseibacillus casei*, have been shown to be important for adaption to nutrient acquisition in environmental niches where the nutrient availability is changing and lost by strains adapted for dairy production [77]. This suggests that the hyaluronate lyase precursor in the examined *L. rhamnosus* strain is related to nutrient acquisition in specific ecological niches and might be a genus-specific trait for the *Lacticaseibacillus* genus.

This exemplifies that annotation errors can lead to misleading conclusions, and further analysis of the genetic context can serve to better understand the function of the gene. Furthermore, this example also supports that the thresholds (80% identity and 70% coverage) provided by EFSA are a good tool to differentiate between genes of potential safety concern and genes that are not considered a safety concern. However, the example above demonstrates the need for expert knowledge and further assessment to make a final conclusion.

## Conclusion

The current study shows the challenges with using databases developed and validated for the clinical area to assess virulence in non-pathogenic bacterial species used for industrial purposes, as the presence of non-pathogenic niche factors in these databases makes it difficult to determine the exact pathogenic potential and may lead to an overestimation of virulence in both non-pathogenic bacteria and pathogenic bacteria. However, applying the EFSA threshold (80% identity and 70% coverage at nucleotide and protein level) serves as a good basis for differentiating between true virulence factors and niche factors.

Multiple hits, with identity and coverage below the EFSA threshold, were found in genes encoding niche factors, but no true virulence factors (e.g. cytotoxic and haemolytic toxins, internalins, invasions, superantigens and neurotoxins) were found in the included industrially relevant species. Furthermore, all the genes were found to be conserved within the species, supporting that the genes are niche factors playing a role in the commensal lifestyle of these non-pathogenic species.

VFDB screening can be used to detect potential virulence factors with identity and coverage above the EFSA threshold. However, a thorough assessment of the gene functionality is needed to determine whether the gene itself can confer virulence or whether it is dependent on other genes, which was the case for the *esx*A gene found in one *S. xylosus* strain.

The lack of true virulence factors in the examined strains correlates with the lack of phenotypic cytotoxicity, haemolysis and negative effect on Caco-2 cell viability. The genotypic and phenotypic assessment showed no indications that the clinical isolates are more virulent than the industrial isolates, which was also supported by the core genome analysis.

Further studies on assessing virulence in non-pathogenic bacteria are needed to differentiate between niche and virulence factors in non-pathogenic bacteria, especially with the increasing use of novel bacterial species for industrial purposes.

## Supplementary material

10.1099/acmi.0.001079.v3 Uncited Supplementary Material 1.

## Abbreviations

ABTS: azino-bis-3-ethylbenzothiazoline-6-sulphonic acid
BHI: brain heart infusion
EFSA: European Food Safety Authority
H_2_O_2_: hydrogen peroxide
LDH: lactate dehydrogenase
MRS: De Man, Rogosa, Sharpe
MTT: 3-(4,5-dimethylthiazol-2-yl)-2,5-diphenyl tetrazolium bromide
QPS: qualified presumption of safety
TSB: Tryptic Soy Broth
VFDB: Virulence Factor Database
