# Supplementary material for: Assessment of pathogenic potential in non-pathogenic industrially relevant bacteria
Source: Access Microbiol. 2026 Jan 30;8(1):001079.v3. doi: 10.1099/acmi.0.001079.v3 (PMC12859219; doi:10.1099/acmi.0.001079.v3)
Supplement: Uncited Supplementary Material 1. [file acmi-8-01079-s001.pdf]

Supplemental material Manuscript III

Supplemental Table S1: VFDB screening at amino acid level for *Staphylococcus xylosus* strain Sx3

Hit	Description	E-value	Query start	%identity	%Gaps
VFG016046(gb	NP_251103) (pvdH) diaminobutyrate-2-oxoglutarate aminotransferase PvdH [pyoverdine (IA001)] [Pseudomonas aeruginosa PAO1]	1.37E-53	12880	31.41	10.55
VFG002158(gb	NP_464456) (lplA1) lipoate protein ligase [LplA1 (VF0347)] [Listeria monocytogenes EGD-e]	1.19E-75	36280	40.74	1.23
VFG001826(gb	NP_217099) (relA) Probable GTP pyrophosphokinase RelA (ATP:GTP 3'-pyrophosphotransferase) (PPGPP synthetase I) ((P)PPGPP synthetase) (GTP diphosphokinase) [RelA (VF0287)] [Mycobacterium tuberculosis H37Rv]	2.29E-178	294404	40.48	1.68
VFG048830(gb	YP_002920353.1) (gnd) 6-phosphogluconate dehydrogenase [Capsule (VF0560)] [Klebsiella pneumoniae subsp. pneumoniae NTUH-K2044]	0.00	411834	65.88	0.21
VFG037028(gb	NP_273273) (katA) catalase [KatA (VF0454)] [Neisseria meningitidis MC58]	0.00	612601	67.90	2.26
VFG002189(gb	NP_816140) (cpsB) phosphatidate cytidylyltransferase [Capsule (VF0361)] [Enterococcus faecalis V583]	3.65E-52	696513	43.98	5.64
VFG002190(gb	NP_816141) (cpsA) undecaprenyl diphosphate synthase [Capsule (VF0361)] [Enterococcus faecalis V583]	3.56E-87	697298	57.69	1.71
VFG037028(gb	NP_273273) (katA) catalase [KatA (VF0454)] [Neisseria meningitidis MC58]	6.77E-104	752202	37.85	4.78
VFG013248(gb	NP_438233) (msbA) lipid transporter ATP-binding/permease [LOS (CVF494)] [Haemophilus influenzae Rd KW20]	4.63E-81	783957	33.73	1.59
VFG007023(gb	NP_231091) (rtxB) RTX toxin transporter RtxB [RTX toxin (CVF263)] [Vibrio cholerae O1 biovar El Tor str. N16961]	1.54E-67	783957	29.49	2.97
VFG000907(gb	NP_755448) (hlyB) Hemolysin B [<alpha>-Hemolysin (VF0225)] [Escherichia coli CFT073]	4.48E-65	783957	29.70	2.15
VFG000841(gb	YP_325609) (hlyB) hemolysin transport protein [Hemolysin (VF0207)] [Escherichia coli O157:H7 str. EDL933]	9.23E-64	783957	31.34	4.71
VFG038916(gb	YP_855895) (rtxB) RTX toxin transporter, ATPase protein [The repeat in toxin (RTX) (CVF795)] [Aeromonas hydrophila subsp. hydrophila ATCC 7966]	3.40E-61	783957	30.31	2.96
VFG038918(gb	YP_855893) (rtxE) RTX toxin transporter, ATPase protein [The repeat in toxin (RTX) (CVF795)] [Aeromonas hydrophila subsp. hydrophila ATCC 7966]	4.12E-60	783957	29.89	6.05
VFG044336(gb	YP_002920268) (iroC) ABC transporter [Sal (VF0563)] [Klebsiella pneumoniae subsp. pneumoniae NTUH-K2044]	9.29E-59	783957	28.34	1.42
VFG000366(gb	NP_405475) (ybtQ) yersiniabactin ABC transporter ATP-binding/permease protein YbtQ [Yersiniabactin (VF0136)] [Yersinia pestis CO92]	5.74E-57	783957	33.64	4.77
VFG044325(gb	YP_002920245) (ybtQ) yersiniabactin ABC transporter ATP-binding/permease protein YbtQ [Ybt (VF0564)] [Klebsiella pneumoniae subsp. pneumoniae NTUH-K2044]	6.16E-57	783957	33.64	4.77
VFG012509(gb	NP_753167) (iroC) ATP binding cassette transporter [Salmochelin (IA013)] [Escherichia coli CFT073]	6.80E-57	783957	27.53	1.42
VFG009579(gb	NP_215865) (irtB) Iron-regulated transporter IrtB [mycobactin (IA031)] [Mycobacterium tuberculosis H37Rv]	4.98E-53	783957	26.71	6.43
VFG000365(gb	NP_405474) (ybtP) yersiniabactin ABC transporter ATP-binding/permease protein YbtP [Yersiniabactin (VF0136)] [Yersinia pestis CO92]	7.77E-51	783957	31.45	6.33
VFG001269(gb	NP_879579) (cyaB) cyclolysin secretion ATP-binding protein [Cya (VF0028)] [Bordetella pertussis Tohama I]	2.28E-72	783960	30.57	0.92
VFG044336(gb	YP_002920268) (iroC) ABC transporter [Sal (VF0563)] [Klebsiella pneumoniae subsp. pneumoniae NTUH-K2044]	5.95E-53	783960	28.20	2.60
VFG002546(gb	YP_109382) (wcbT) acyl-CoA transferase [Capsule I (VF0436)] [Burkholderia pseudomallei K96243]	6.13E-57	817833	33.25	2.58
VFG046465(gb	YP_169203.1) (tufA) elongation factor Tu [EF-Tu (VF0460)] [Francisella tularensis subsp. tularensis SCHU S4]	6.62E-171	820681	73.42	0.26
VFG002076(gb	NP_248780) (clpV1) type VI secretion system AAA+ family ATPase [HSI-I (VF0334)] [Pseudomonas aeruginosa PAO1]	2.87E-61	847784	38.76	4.14
VFG000080(gb	NP_464522) (clpE) ATP-dependent protease [ClpE (VF0073)] [Listeria monocytogenes EGD-e]	0.00	847802	51.32	4.19
VFG000079(gb	NP_463763) (clpC) endopeptidase Clp ATP-binding chain C [ClpC (VF0072)] [Listeria monocytogenes EGD-e]	0.00	847808	70.38	0.25
VFG002480(gb	YP_111509) (tssH-5/clpV) Clp-type ATPase chaperone protein [T6SS-1 (VF0429)] [Burkholderia pseudomallei K96243]	3.28E-65	847850	41.21	2.88
VFG048693(gb	YP_005226603.1) (clpV/tssH) type VI secretion system ATPase TssH [T6SS (VF0569)] [Klebsiella pneumoniae subsp. pneumoniae HS11286]	6.52E-148	847859	40.39	7.11
VFG038395(gb	YP_856375) (clpB) type VI secretion system ATPase ClpV1 [T6SS (SS194)] [Aeromonas hydrophila subsp. hydrophila ATCC 7966]	4.64E-135	847910	35.55	11.18
VFG002084(gb	NP_232517) (clpB/vasG) type VI secretion system AAA+ family ATPase [T6SS (VF0335)] [Vibrio cholerae O1 biovar El Tor str. N16961]	1.84E-126	847910	38.79	10.45
VFG049904(gb	YP_309261.1) (clpV/tssH) Type VI secretion system ATPase ClpV/TssH [T6SS (VF0579)] [Shigella sonnei Ss046]	7.09E-51	847910	39.52	4.12
VFG049904(gb	YP_309261.1) (clpV/tssH) Type VI secretion system ATPase ClpV/TssH [T6SS (VF0579)] [Shigella sonnei Ss046]	5.77E-71	848840	48.46	2.73
VFG002076(gb	NP_248780) (clpV1) type VI secretion system AAA+ family ATPase [HSI-I (VF0334)] [Pseudomonas aeruginosa PAO1]	3.76E-64	848840	45.33	4.33
VFG002480(gb	YP_111509) (tssH-5/clpV) Clp-type ATPase chaperone protein [T6SS-1 (VF0429)] [Burkholderia pseudomallei K96243]	8.83E-73	848918	53.82	0.38
VFG002361(gb	YP_001007253) (galE) UDP-glucose 4-epimerase [O-antigen (VF0392)] [Yersinia enterocolitica subsp. enterocolitica 8081]	1.05E-74	931392	41.76	5.00
VFG013286(gb	NP_438515) (galE) UDP-glucose 4-epimerase [LOS (CVF494)] [Haemophilus influenzae Rd KW20]	1.26E-71	931392	39.94	3.25
VFG046645(gb	YP_169798.1) (galE) UDP-glucose 4-epimerase GalE [Capsule (VF0543)] [Francisella tularensis subsp. tularensis SCHU S4]	1.72E-71	931416	43.90	3.35
VFG002188(gb	NP_816139) (cpsC) teichoic acid biosynthesis protein, putative [Capsule (VF0361)] [Enterococcus faecalis V583]	2.06E-77	1089301	39.24	4.63
VFG002188(gb	NP_816139) (cpsC) teichoic acid biosynthesis protein, putative [Capsule (VF0361)] [Enterococcus faecalis V583]	7.77E-87	1091568	42.23	3.27
VFG004773(gb	NP_647407) (lip) triacylglycerol lipase precursor [Lipase (CVF091)] [Staphylococcus aureus subsp. aureus MW2]	1.82E-126	1208462	50.00	0.79
VFG001316(gb	NP_645114) (geh) glycerol ester hydrolase [Lipase (VF0012)] [Staphylococcus aureus subsp. aureus MW2]	2.67E-118	1208468	47.16	0.52
VFG002405(gb	NP_645073) (esxA) type VII secretion system secreted protein EsxA [Type VII secretion system (VF0403)] [Staphylococcus aureus subsp. aureus MW2]	8.36E-53	1259395	94.85	0.00
VFG002406(gb	NP_645074) (esaA) type VII secretion system protein EsaA [Type VII secretion system (VF0403)] [Staphylococcus aureus subsp. aureus MW2]	0.00	1259775	56.12	1.57

VFG002408(gb	NP_645078) (essB) type VII secretion system protein EssB, monotopic membrane protein [Type VII secretion system (VF0403)] [Staphylococcus aureus subsp. aureus MW2]	4.09E-180	1263551	71.84	0.53
VFG002409(gb	NP_645079) (essC) type VII secretion system protein EssC, FtsK/SpoIIIE family ATPase [Type VII secretion system (VF0403)] [Staphylococcus aureus subsp. aureus MW2]	0.00	1264870	67.07	0.34
VFG040864(gb	YP_028298) (essC) type VII secretion system protein EssC [T7SS (VF0581)] [Bacillus anthracis str. Sterne]	3.12E-152	1265470	30.38	4.74
VFG000670(gb	NP_706258) (gtrB) bactoprenol glucosyl transferase [LPS (VF0124)] [Shigella flexneri 2a str. 301]	1.65E-66	1289949	41.88	1.95
VFG046903(gb	YP_169492.1) (flmF2) glycosyl transferase family protein [LPS (VF0542)] [Francisella tularensis subsp. tularensis SCHU S4]	8.90E-58	1289949	38.56	1.96
VFG002179(gb	AAD09858) (esp) Enterococcal surface protein; Esp [Esp (VF0353)] [Enterococcus faecalis str. MMH594]	1.12E-58	1373576	33.54	11.88
VFG049120(gb	YP_002918207.1) (allB) allantoinase [Allantion utilization (VF0572)] [Klebsiella pneumoniae subsp. pneumoniae NTUH-K2044]	4.48E-171	1393903	56.29	1.10
VFG049122(gb	YP_002918211.1) (allC) allantoate amidohydrolase [Allantion utilization (VF0572)] [Klebsiella pneumoniae subsp. pneumoniae NTUH-K2044]	8.20E-123	1399516	53.49	0.78
VFG049124(gb	YP_002918212.1) (allD) ureidoglycolate dehydrogenase [Allantion utilization (VF0572)] [Klebsiella pneumoniae subsp. pneumoniae NTUH-K2044]	1.96E-129	1401590	57.31	0.00
VFG049124(gb	YP_002918212.1) (allD) ureidoglycolate dehydrogenase [Allantion utilization (VF0572)] [Klebsiella pneumoniae subsp. pneumoniae NTUH-K2044]	8.60E-136	1402759	57.59	0.00
VFG004773(gb	NP_647407) (lip) triacylglycerol lipase precursor [Lipase (CVF091)] [Staphylococcus aureus subsp. aureus MW2]	0.00	1423467	52.90	14.30
VFG001316(gb	NP_645114) (geh) glycerol ester hydrolase [Lipase (VF0012)] [Staphylococcus aureus subsp. aureus MW2]	3.38E-141	1424307	51.16	2.79
VFG001359(gb	NP_346089) (psaA) manganese ABC transporter, manganese-binding adhesion liprotein [PsaA (VF0151)] [Streptococcus pneumoniae TIGR4]	5.81E-56	1471329	39.46	3.34
VFG002165(gb	NP_815739) (efaA) endocarditis specific antigen [EfaA (VF0354)] [Enterococcus faecalis V583]	1.26E-55	1471383	40.14	0.72
VFG002157(gb	NP_465372) (lpeA) lipoprotein promoting cell invasion [LpeA (VF0346)] [Listeria monocytogenes EGD-e]	1.54E-63	1471389	42.70	1.78
VFG001269(gb	NP_879579) (cyaB) cyclolysin secretion ATP-binding protein [Cya (VF0028)] [Bordetella pertussis Tohama I]	1.95E-65	1704601	29.36	3.91
VFG038918(gb	YP_855893) (rtxE) RTX toxin transporter, ATPase protein [The repeat in toxin (RTX) (CVF795)] [Aeromonas hydrophila subsp. hydrophila ATCC 7966]	8.57E-57	1704601	28.67	2.15
VFG009570(gb	NP_215864) (irtA) Iron-regulated transporter IrtA [mycobactin (IA031)] [Mycobacterium tuberculosis H37Rv]	4.90E-55	1704601	29.84	7.00
VFG000907(gb	NP_755448) (hlyB) Hemolysin B [<alpha>-Hemolysin (VF0225)] [Escherichia coli CFT073]	1.45E-65	1704604	31.63	6.50
VFG038916(gb	YP_855895) (rtxB) RTX toxin transporter, ATPase protein [The repeat in toxin (RTX) (CVF795)] [Aeromonas hydrophila subsp. hydrophila ATCC 7966]	3.49E-54	1704604	27.40	7.83
VFG013248(gb	NP_438233) (msbA) lipid transporter ATP-binding/permease [LOS (CVF494)] [Haemophilus influenzae Rd KW20]	3.99E-76	1704607	30.50	3.47
VFG007023(gb	NP_231091) (rtxB) RTX toxin transporter RtxB [RTX toxin (CVF263)] [Vibrio cholerae O1 biovar El Tor str. N16961]	9.04E-59	1704607	28.34	4.69
VFG000841(gb	YP_325609) (hlyB) hemolysin transport protein [Hemolysin (VF0207)] [Escherichia coli O157:H7 str. EDL933]	2.36E-58	1704607	30.54	7.14
VFG012509(gb	NP_753167) (iroC) ATP binding cassette transporter [Salmochelin (IA013)] [Escherichia coli CFT073]	4.92E-60	1704616	27.96	3.01
VFG044336(gb	YP_002920268) (iroC) ABC transporter [Sal (VF0563)] [Klebsiella pneumoniae subsp. pneumoniae NTUH-K2044]	3.83E-58	1704616	27.08	3.01
VFG000365(gb	NP_405474) (ybtP) yersiniabactin ABC transporter ATP-binding/permease protein YbtP [Yersiniabactin (VF0136)] [Yersinia pestis CO92]	1.61E-53	1704616	28.33	8.17
VFG044326(gb	YP_002920246) (ybtP) yersiniabactin ABC transporter ATP-binding/permease protein YbtP [Ybt (VF0564)] [Klebsiella pneumoniae subsp. pneumoniae NTUH-K2044]	1.76E-53	1704616	28.14	8.17
VFG044336(gb	YP_002920268) (iroC) ABC transporter [Sal (VF0563)] [Klebsiella pneumoniae subsp. pneumoniae NTUH-K2044]	1.75E-54	1704649	27.07	8.29
VFG001269(gb	NP_879579) (cyaB) cyclolysin secretion ATP-binding protein [Cya (VF0028)] [Bordetella pertussis Tohama I]	5.81E-61	1706414	31.73	3.21
VFG000841(gb	YP_325609) (hlyB) hemolysin transport protein [Hemolysin (VF0207)] [Escherichia coli O157:H7 str. EDL933]	1.06E-66	1706423	32.87	1.40
VFG000907(gb	NP_755448) (hlyB) Hemolysin B [<alpha>-Hemolysin (VF0225)] [Escherichia coli CFT073]	2.71E-58	1706423	30.93	3.30
VFG013248(gb	NP_438233) (msbA) lipid transporter ATP-binding/permease [LOS (CVF494)] [Haemophilus influenzae Rd KW20]	9.07E-67	1706426	30.85	3.23
VFG007023(gb	NP_231091) (rtxB) RTX toxin transporter RtxB [RTX toxin (CVF263)] [Vibrio cholerae O1 biovar El Tor str. N16961]	5.91E-55	1706426	31.57	3.67
VFG000679(gb	AAF13660) (dep/capD) gamma-glutamyltranspeptidase, required for polyglutamate anchoring to peptidoglycan [Capsule (VF0141)] [Bacillus anthracis]	2.20E-56	1742587	33.19	4.34
VFG046835(gb	YP_169812.1) (capB) capsule biosynthesis protein CapB [Capsule (VF0543)] [Francisella tularensis subsp. tularensis SCHU S4]	5.33E-54	1745851	37.77	3.46
VFG000682(gb	AAF13663) (capB) CapB, involved in Poly-gamma-glutamate synthesis [Capsule (VF0141)] [Bacillus anthracis]	2.85E-109	1745854	50.55	0.55
VFG002165(gb	NP_815739) (efaA) endocarditis specific antigen [EfaA (VF0354)] [Enterococcus faecalis V583]	1.51E-89	1844241	52.05	2.40
VFG002157(gb	NP_465372) (lpeA) lipoprotein promoting cell invasion [LpeA (VF0346)] [Listeria monocytogenes EGD-e]	8.67E-86	1844241	50.00	1.03
VFG001359(gb	NP_346089) (psaA) manganese ABC transporter, manganese-binding adhesion liprotein [PsaA (VF0151)] [Streptococcus pneumoniae TIGR4]	5.74E-79	1844253	46.18	1.74
VFG002181(gb	NP_816132) (cpsJ) ABC transporter, ATP-binding protein [Capsule (VF0361)] [Enterococcus faecalis V583]	5.19E-72	1849664	51.42	0.40
VFG049084(gb	YP_002920347.1) (wzt) lipopolysaccharide O-antigen ABC transport system ATP-binding component [LPS (VF0561)] [Klebsiella pneumoniae subsp. pneumoniae NTUH-K2044]	1.30E-54	1849715	38.87	4.45
VFG013248(gb	NP_438233) (msbA) lipid transporter ATP-binding/permease [LOS (CVF494)] [Haemophilus influenzae Rd KW20]	2.43E-86	1856457	32.94	4.78
VFG000841(gb	YP_325609) (hlyB) hemolysin transport protein [Hemolysin (VF0207)] [Escherichia coli O157:H7 str. EDL933]	3.63E-61	1856508	30.33	3.12
VFG000907(gb	NP_755448) (hlyB) Hemolysin B [<alpha>-Hemolysin (VF0225)] [Escherichia coli CFT073]	1.01E-68	1856511	30.70	4.49
VFG001269(gb	NP_879579) (cyaB) cyclolysin secretion ATP-binding protein [Cya (VF0028)] [Bordetella pertussis Tohama I]	6.02E-66	1856523	29.66	2.71
VFG007023(gb	NP_231091) (rtxB) RTX toxin transporter RtxB [RTX toxin (CVF263)] [Vibrio cholerae O1 biovar El Tor str. N16961]	3.02E-56	1856547	27.27	2.18
VFG038916(gb	YP_855895) (rtxB) RTX toxin transporter, ATPase protein [The repeat in toxin (RTX) (CVF795)] [Aeromonas hydrophila subsp. hydrophila ATCC 7966]	3.45E-52	1856553	28.26	3.30
VFG000365(gb	NP_405474) (ybtP) yersiniabactin ABC transporter ATP-binding/permease protein YbtP [Yersiniabactin (VF0136)] [Yersinia pestis CO92]	9.57E-52	1856721	28.16	5.92
VFG044326(gb	YP_002920246) (ybtP) yersiniabactin ABC transporter ATP-binding/permease protein YbtP [Ybt (VF0564)] [Klebsiella pneumoniae subsp. pneumoniae NTUH-K2044]	9.57E-52	1856721	28.16	5.92
VFG009570(gb	NP_215864) (irtA) Iron-regulated transporter IrtA [mycobactin (IA031)] [Mycobacterium tuberculosis H37Rv]	1.17E-51	1856766	30.04	5.67

VFG048488(gb	YP_002918371.1) (fepC) iron-enterobactin transporter ATP-binding protein [Ent (VF0562)] [Klebsiella pneumoniae subsp. pneumoniae NTUH-K2044]	1.30E-82	1861536	48.64	0.00
VFG000925(gb	NP_752606) (fepC) ferrienterobactin ABC transporter ATPase [Enterobactin (VF0228)] [Escherichia coli CFT073]	5.11E-81	1861548	49.01	0.00
VFG037386(gb	YP_001847242) (bauE) ferric siderophore ABC transporter, ATP-binding protein BauE [Acinetobactin (VF0467)] [Acinetobacter baumannii ACICU]	1.10E-54	1861575	41.77	0.84
VFG000964(gb	NP_270109) (hasC) UDP-glucose pyrophosphorylase [Hyaluronic acid capsule (VF0244)] [Streptococcus pyogenes M1 GAS]	8.25E-84	1910579	52.58	2.06
VFG013346(gb	NP_438972) (galU) glucosephosphate uridylyltransferase [LOS (CVF494)] [Haemophilus influenzae Rd KW20]	1.19E-61	1910594	42.76	3.10
VFG048990(gb	YP_002920369.1) (galF) UTP-glucose-1-phosphate uridylyltransferase subunit GalF [Capsule (VF0560)] [Klebsiella pneumoniae subsp. pneumoniae NTUH-K2044]	4.26E-54	1910594	41.52	3.97
VFG000670(gb	NP_706258) (gtrB) bactoprenol glucosyl transferase [LPS (VF0124)] [Shigella flexneri 2a str. 301]	1.58E-60	1920534	43.09	2.25
VFG046903(gb	YP_169492.1) (flmF2) glycosyl transferase family protein [LPS (VF0542)] [Francisella tularensis subsp. tularensis SCHU S4]	7.29E-54	1920537	36.96	4.66
VFG037414(gb	YP_001847244) (bauD) ferric siderophore ABC transporter, permease protein BauD [Acinetobactin (VF0467)] [Acinetobacter baumannii ACICU]	4.30E-60	1951536	38.16	0.00
VFG037386(gb	YP_001847242) (bauE) ferric siderophore ABC transporter, ATP-binding protein BauE [Acinetobactin (VF0467)] [Acinetobacter baumannii ACICU]	1.06E-66	1953378	47.66	0.00
VFG000077(gb	NP_465991) (clpP) ATP-dependent Clp protease proteolytic subunit [ClpP (VF0074)] [Listeria monocytogenes EGD-e]	9.15E-98	1993334	77.32	0.00
VFG032992(gb	NP_464816) (oatA) peptidoglycan O-acetyltransferase [OatA (VF0441)] [Listeria monocytogenes EGD-e]	2.09E-92	2123945	36.96	5.01
VFG000079(gb	NP_463763) (clpC) endopeptidase Clp ATP-binding chain C [ClpC (VF0072)] [Listeria monocytogenes EGD-e]	0.00	2126234	44.78	9.99
VFG038395(gb	YP_856375) (clpB) type VI secretion system ATPase ClpV1 [T6SS (SS194)] [Aeromonas hydrophila subsp. hydrophila ATCC 7966]	4.35E-150	2126657	40.17	5.06
VFG000080(gb	NP_464522) (clpE) ATP-dependent protease [ClpE (VF0073)] [Listeria monocytogenes EGD-e]	0.00	2126666	46.56	11.36
VFG048693(gb	YP_005226603.1) (clpV/tssH) type VI secretion system ATPase TssH [T6SS (VF0569)] [Klebsiella pneumoniae subsp. pneumoniae HS11286]	1.65E-154	2126681	40.35	5.33
VFG049904(gb	YP_309261.1) (clpV/tssH) Type VI secretion system ATPase ClpV/TssH [T6SS (VF0579)] [Shigella sonnei Ss046]	3.24E-137	2126687	39.76	4.97
VFG002076(gb	NP_248780) (clpV1) type VI secretion system AAA+ family ATPase [HSI-I (VF0334)] [Pseudomonas aeruginosa PAO1]	6.29E-147	2126699	42.63	3.61
VFG002480(gb	YP_111509) (tssH-5/clpV) Clp-type ATPase chaperone protein [T6SS-1 (VF0429)] [Burkholderia pseudomallei K96243]	1.04E-80	2126702	50.00	0.95
VFG002084(gb	NP_232517) (clpB/vasG) type VI secretion system AAA+ family ATPase [T6SS (VF0335)] [Vibrio cholerae O1 biovar El Tor str. N16961]	3.39E-139	2126705	39.80	5.21
VFG002480(gb	YP_111509) (tssH-5/clpV) Clp-type ATPase chaperone protein [T6SS-1 (VF0429)] [Burkholderia pseudomallei K96243]	1.63E-68	2127782	40.12	1.54
VFG002158(gb	NP_464456) (lplA1) lipoate protein ligase [LplA1 (VF0347)] [Listeria monocytogenes EGD-e]	5.32E-111	2174095	52.73	0.91
VFG000078(gb	NP_466081) (ami) autolysin amidase, adhesin [Ami (VF0071)] [Listeria monocytogenes EGD-e]	1.02E-54	2191741	57.38	1.09
VFG013327(gb	NP_438900) (yhxB/manB) phosphomannomutase [LOS (CVF494)] [Haemophilus influenzae Rd KW20]	3.84E-60	2196928	31.12	9.76
VFG047564(gb	YP_169890.1) (purM) phosphoribosylaminoimidazole synthetase [Purine biosynthesis (VF0559)] [Francisella tularensis subsp. tularensis SCHU S4]	1.15E-77	2215226	42.73	4.45
VFG047582(gb	YP_169891.1) (purCD) fusion protein PurC/PurD [Purine biosynthesis (VF0559)] [Francisella tularensis subsp. tularensis SCHU S4]	6.11E-51	2218326	32.03	4.61
VFG047726(gb	YP_170570.1) (carA) carbamoyl phosphate synthase small subunit [Pyrimidine biosynthesis (VF0558)] [Francisella tularensis subsp. tularensis SCHU S4]	3.88E-95	2339073	47.47	6.40
VFG047708(gb	YP_170571.1) (carB) carbamoyl phosphate synthase large subunit [Pyrimidine biosynthesis (VF0558)] [Francisella tularensis subsp. tularensis SCHU S4]	0.00	2340148	47.83	3.69
VFG000959(gb	NP_269190) (fbp54) fibronectin-bing protein Fbp54 [FBPs (VF0243)] [Streptococcus pyogenes M1 GAS]	5.41E-121	2347196	42.25	4.05
VFG005177(gb	NP_664456) (fbp54) fibronectin-bing protein FbaA [Fibronectin-binding proteins (CVF113)] [Streptococcus pyogenes MGAS315]	6.03E-119	2347196	41.73	4.05
VFG002160(gb	NP_465354) (fbpA) fibronectin-binding protein [FbpA (VF0349)] [Listeria monocytogenes EGD-e]	1.08E-145	2347199	43.06	1.05
VFG005197(gb	NP_358462) (pavA) Fibronectin-binding protein-like protein A [PavA (VF0283)] [Streptococcus pneumoniae R6]	1.69E-116	2347199	40.03	6.93
VFG001314(gb	NP_647375) (aur) zinc metalloproteinase aureolysin [Aureolysin (VF0024)] [Staphylococcus aureus subsp. aureus MW2]	1.33E-130	2371217	47.68	4.44
VFG000071(gb	NP_463734) (mpl) Zinc metalloproteinase precursor [Mpl (VF0065)] [Listeria monocytogenes EGD-e]	7.85E-103	2371220	43.01	3.49
VFG005767(gb	NP_687682) (cylG) 3-ketoacyl-ACP-reductase CylG [Beta-hemolysin/cytolysin (CVF171)] [Streptococcus agalactiae 2603V/R]	1.24E-55	2375705	43.80	4.96
VFG038840(gb	YP_008043465) (flmH) flagellar-related 3-oxoacyl-ACP reductase [Polar flagella (VF0473)] [Aeromonas hydrophila ML09-119]	3.15E-71	2375708	50.63	1.67
VFG001283(gb	NP_647238) (fnbA) fibronectin-binding protein A [FnBPs (VF0010)] [Staphylococcus aureus subsp. aureus MW2]	3.63E-79	2384716	36.41	11.78
VFG001282(gb	NP_647237) (fnbB) fibronectin-binding protein B [FnBPs (VF0010)] [Staphylococcus aureus subsp. aureus MW2]	1.60E-73	2384716	33.64	13.12
VFG001312(gb	NP_644954) (cap8P) capsular polysaccharide synthesis enzyme Cap8P [Capsule (VF0003)] [Staphylococcus aureus subsp. aureus MW2]	3.75E-159	2474808	61.60	0.00
VFG001373(gb	NP_344890) (cps4I) UDP-N-acetylglucosamine-2-epimerase [Capsule (VF0144)] [Streptococcus pneumoniae TIGR4]	4.73E-145	2474808	59.33	0.00
VFG000274(gb	NP_206868) (ureG) urease accessory protein (ureG) [Urease (VF0050)] [Helicobacter pylori 26695]	1.33E-78	2541130	60.91	0.00
VFG000270(gb	NP_206872) (ureB) urease beta subunit UreB, urea amidohydrolase [Urease (VF0050)] [Helicobacter pylori 26695]	0.00	2542894	57.37	0.88
VFG000269(gb	NP_206873) (ureA) urease alpha subunit UreA [Urease (VF0050)] [Helicobacter pylori 26695]	5.91E-68	2544711	56.52	1.45
VFG002188(gb	NP_816139) (cpsC) teichoic acid biosynthesis protein, putative [Capsule (VF0361)] [Enterococcus faecalis V583]	5.13E-84	2583308	40.49	2.45
VFG000907(gb	NP_755448) (hlyB) Hemolysin B [<alpha>-Hemolysin (VF0225)] [Escherichia coli CFT073]	1.56E-92	2728179	34.76	1.88
VFG007023(gb	NP_231091) (rtxB) RTX toxin transporter RtxB [RTX toxin (CVF263)] [Vibrio cholerae O1 biovar El Tor str. N16961]	1.32E-82	2728179	32.33	0.80
VFG038916(gb	YP_855895) (rtxB) RTX toxin transporter, ATPase protein [The repeat in toxin (RTX) (CVF795)] [Aeromonas hydrophila subsp. hydrophila ATCC 7966]	5.11E-78	2728179	33.01	0.78
VFG038918(gb	YP_855893) (rtxE) RTX toxin transporter, ATPase protein [The repeat in toxin (RTX) (CVF795)] [Aeromonas hydrophila subsp. hydrophila ATCC 7966]	1.32E-71	2728179	33.01	1.54
VFG001269(gb	NP_879579) (cyaB) cyclolysin secretion ATP-binding protein [Cya (VF0028)] [Bordetella pertussis Tohama I]	2.53E-96	2728182	37.60	1.80
VFG000841(gb	YP_325609) (hlyB) hemolysin transport protein [Hemolysin (VF0207)] [Escherichia coli O157:H7 str. EDL933]	8.98E-96	2728182	38.83	0.80

VFG013248(gb	NP_438233) (msbA) lipid transporter ATP-binding/permease [LOS (CVF494)] [Haemophilus influenzae Rd KW20]	2.57E-87	2728182	33.66	1.16
VFG000366(gb	NP_405475) (ybtQ) yersiniabactin ABC transporter ATP-binding/permease protein YbtQ [Yersiniabactin (VF0136)] [Yersinia pestis CO92]	2.48E-71	2728182	32.51	4.56
VFG044325(gb	YP_002920245) (ybtQ) yersiniabactin ABC transporter ATP-binding/permease protein YbtQ [Ybt (VF0564)] [Klebsiella pneumoniae subsp. pneumoniae NTUH-K2044]	2.85E-71	2728182	32.51	4.56
VFG044336(gb	YP_002920268) (iroC) ABC transporter [Sal (VF0563)] [Klebsiella pneumoniae subsp. pneumoniae NTUH-K2044]	1.81E-65	2728188	31.88	3.37
VFG012509(gb	NP_753167) (iroC) ATP binding cassette transporter [Salmochelin (IA013)] [Escherichia coli CFT073]	2.84E-62	2728188	31.87	1.99
VFG000365(gb	NP_405474) (ybtP) yersiniabactin ABC transporter ATP-binding/permease protein YbtP [Yersiniabactin (VF0136)] [Yersinia pestis CO92]	4.19E-63	2728191	33.06	4.90
VFG044326(gb	YP_002920246) (ybtP) yersiniabactin ABC transporter ATP-binding/permease protein YbtP [Ybt (VF0564)] [Klebsiella pneumoniae subsp. pneumoniae NTUH-K2044]	1.88E-62	2728191	32.86	4.90
VFG012509(gb	NP_753167) (iroC) ATP binding cassette transporter [Salmochelin (IA013)] [Escherichia coli CFT073]	2.24E-61	2728191	32.80	2.60
VFG044336(gb	YP_002920268) (iroC) ABC transporter [Sal (VF0563)] [Klebsiella pneumoniae subsp. pneumoniae NTUH-K2044]	5.26E-60	2728191	32.13	1.81
VFG009570(gb	NP_215864) (irtA) Iron-regulated transporter IrtA [mycobactin (IA031)] [Mycobacterium tuberculosis H37Rv]	1.81E-51	2728191	44.87	0.85
VFG001855(gb	YP_094724) (htpB) Hsp60, 60K heat shock protein HtpB [Hsp60 (VF0159)] [Legionella pneumophila subsp. pneumophila str. Philadelphia 1]	5.24E-162	2797824	55.89	0.38
VFG001403(gb	NP_216899) (mbtB) Phenyloxazoline synthase MbtB (phenyloxazoline synthetase) [Mycobactin (VF0299)] [Mycobacterium tuberculosis H37Rv]	1.40E-71	2838588	24.09	7.97
VFG049160(gb	YP_006635486.1) (clbN) colibactin non-ribosomal peptide synthetase ClbN [Colibactin (VF0573)] [Klebsiella pneumoniae subsp. pneumoniae 1084]	1.99E-65	2838663	24.44	6.25
VFG016041(gb	NP_251092) (pvdI) peptide synthase [pyoverdine (IA001)] [Pseudomonas aeruginosa PAO1]	1.32E-109	2838774	28.16	5.80
VFG016058(gb	NP_251114) (pvdL) peptide synthase PvdL [pyoverdine (IA001)] [Pseudomonas aeruginosa PAO1]	0.00	2838783	28.81	10.69
VFG016038(gb	NP_251090) (pvdJ) pyoverdine biosynthesis protein PvdJ [pyoverdine (IA001)] [Pseudomonas aeruginosa PAO1]	0.00	2838789	32.55	5.29
VFG016058(gb	NP_251114) (pvdL) peptide synthase PvdL [pyoverdine (IA001)] [Pseudomonas aeruginosa PAO1]	3.94E-123	2838804	28.28	8.78
VFG000930(gb	NP_752604) (entF) enterobactin synthase multienzyme complex component, ATP-dependent [Enterobactin (VF0228)] [Escherichia coli CFT073]	1.00E-98	2838804	26.48	8.86
VFG001821(gb	NP_216896) (mbtE) Peptide synthetase MbtE (peptide synthase) [Mycobactin (VF0299)] [Mycobacterium tuberculosis H37Rv]	7.00E-131	2838813	29.73	5.46
VFG016058(gb	NP_251114) (pvdL) peptide synthase PvdL [pyoverdine (IA001)] [Pseudomonas aeruginosa PAO1]	0.00	2838828	30.30	7.58
VFG049156(gb	YP_006635482.1) (clbJ) colibactin non-ribosomal peptide synthetase ClbJ [Colibactin (VF0573)] [Klebsiella pneumoniae subsp. pneumoniae 1084]	8.81E-112	2838828	27.93	5.82
VFG016041(gb	NP_251092) (pvdI) peptide synthase [pyoverdine (IA001)] [Pseudomonas aeruginosa PAO1]	1.41E-110	2838828	28.64	7.84
VFG049154(gb	YP_006635480.1) (clbH) colibactin non-ribosomal peptide synthetase ClbH [Colibactin (VF0573)] [Klebsiella pneumoniae subsp. pneumoniae 1084]	8.10E-117	2838837	26.40	4.01
VFG001820(gb	NP_216895) (mbtF) Peptide synthetase MbtF (peptide synthase) [Mycobactin (VF0299)] [Mycobacterium tuberculosis H37Rv]	6.93E-75	2838837	25.54	8.02
VFG000161(gb	NP_251089) (pvdD) pyoverdine synthetase D [Pyoverdine (VF0094)] [Pseudomonas aeruginosa PAO1]	0.00	2838843	34.51	4.61
VFG048498(gb	YP_002918370.1) (entF) enterobactin synthase subunit F [Ent (VF0562)] [Klebsiella pneumoniae subsp. pneumoniae NTUH-K2044]	1.39E-99	2838843	32.31	6.43
VFG049156(gb	YP_006635482.1) (clbJ) colibactin non-ribosomal peptide synthetase ClbJ [Colibactin (VF0573)] [Klebsiella pneumoniae subsp. pneumoniae 1084]	2.81E-73	2838843	29.12	6.57
VFG049148(gb	YP_006635474.1) (clbB) colibactin hybrid non-ribosomal peptide synthetase/type I polyketide synthase ClbB [Colibactin (VF0573)] [Klebsiella pneumoniae subsp. pneumoniae 1084]	4.05E-62	2838855	25.58	7.01
VFG016041(gb	NP_251092) (pvdI) peptide synthase [pyoverdine (IA001)] [Pseudomonas aeruginosa PAO1]	0.00	2838858	33.88	4.07
VFG016041(gb	NP_251092) (pvdI) peptide synthase [pyoverdine (IA001)] [Pseudomonas aeruginosa PAO1]	0.00	2838870	37.09	4.53
VFG049154(gb	YP_006635480.1) (clbH) colibactin non-ribosomal peptide synthetase ClbH [Colibactin (VF0573)] [Klebsiella pneumoniae subsp. pneumoniae 1084]	3.26E-95	2839092	38.01	4.68
VFG049157(gb	YP_006635483.1) (clbK) colibactin hybrid non-ribosomal peptide synthetase/type I polyketide synthase ClbK [Colibactin (VF0573)] [Klebsiella pneumoniae subsp. pneumoniae 1084]	2.61E-72	2839275	24.24	8.28
VFG000165(gb	NP_252915) (pchF) pyochelin synthetase PchF [Pyochelin (VF0095)] [Pseudomonas aeruginosa PAO1]	8.09E-57	2839311	31.91	8.13
VFG001821(gb	NP_216896) (mbtE) Peptide synthetase MbtE (peptide synthase) [Mycobactin (VF0299)] [Mycobacterium tuberculosis H37Rv]	0.00	2840607	28.69	5.19
VFG001820(gb	NP_216895) (mbtF) Peptide synthetase MbtF (peptide synthase) [Mycobactin (VF0299)] [Mycobacterium tuberculosis H37Rv]	2.20E-77	2840991	23.70	9.57
VFG016058(gb	NP_251114) (pvdL) peptide synthase PvdL [pyoverdine (IA001)] [Pseudomonas aeruginosa PAO1]	0.00	2841000	29.38	8.29
VFG016041(gb	NP_251092) (pvdI) peptide synthase [pyoverdine (IA001)] [Pseudomonas aeruginosa PAO1]	0.00	2841000	31.43	6.74
VFG016041(gb	NP_251092) (pvdI) peptide synthase [pyoverdine (IA001)] [Pseudomonas aeruginosa PAO1]	0.00	2841000	33.50	5.43
VFG048498(gb	YP_002918370.1) (entF) enterobactin synthase subunit F [Ent (VF0562)] [Klebsiella pneumoniae subsp. pneumoniae NTUH-K2044]	2.65E-106	2841921	27.43	8.01
VFG000161(gb	NP_251089) (pvdD) pyoverdine synthetase D [Pyoverdine (VF0094)] [Pseudomonas aeruginosa PAO1]	0.00	2841924	34.69	3.52
VFG049156(gb	YP_006635482.1) (clbJ) colibactin non-ribosomal peptide synthetase ClbJ [Colibactin (VF0573)] [Klebsiella pneumoniae subsp. pneumoniae 1084]	0.00	2841924	25.95	7.07
VFG001403(gb	NP_216899) (mbtB) Phenyloxazoline synthase MbtB (phenyloxazoline synthetase) [Mycobactin (VF0299)] [Mycobacterium tuberculosis H37Rv]	5.22E-63	2841933	26.01	8.49
VFG016041(gb	NP_251092) (pvdI) peptide synthase [pyoverdine (IA001)] [Pseudomonas aeruginosa PAO1]	1.09E-126	2841939	30.43	6.67
VFG000930(gb	NP_752604) (entF) enterobactin synthase multienzyme complex component, ATP-dependent [Enterobactin (VF0228)] [Escherichia coli CFT073]	1.46E-101	2841939	30.89	4.76
VFG049148(gb	YP_006635474.1) (clbB) colibactin hybrid non-ribosomal peptide synthetase/type I polyketide synthase ClbB [Colibactin (VF0573)] [Klebsiella pneumoniae subsp. pneumoniae 1084]	3.08E-77	2841954	28.37	6.74
VFG049154(gb	YP_006635480.1) (clbH) colibactin non-ribosomal peptide synthetase ClbH [Colibactin (VF0573)] [Klebsiella pneumoniae subsp. pneumoniae 1084]	1.24E-102	2841957	28.93	3.31
VFG016038(gb	NP_251090) (pvdJ) pyoverdine biosynthesis protein PvdJ [pyoverdine (IA001)] [Pseudomonas aeruginosa PAO1]	0.00	2841969	32.85	4.48
VFG016058(gb	NP_251114) (pvdL) peptide synthase PvdL [pyoverdine (IA001)] [Pseudomonas aeruginosa PAO1]	1.53E-132	2841969	30.22	8.81
VFG049160(gb	YP_006635486.1) (clbN) colibactin non-ribosomal peptide synthetase ClbN [Colibactin (VF0573)] [Klebsiella pneumoniae subsp. pneumoniae 1084]	9.31E-79	2842146	25.00	9.27
VFG049154(gb	YP_006635480.1) (clbH) colibactin non-ribosomal peptide synthetase ClbH [Colibactin (VF0573)] [Klebsiella pneumoniae subsp. pneumoniae 1084]	2.73E-88	2842149	35.86	4.17
VFG049157(gb	YP_006635483.1) (clbK) colibactin hybrid non-ribosomal peptide synthetase/type I polyketide synthase ClbK [Colibactin (VF0573)] [Klebsiella pneumoniae subsp. pneumoniae 1084]	2.93E-62	2842389	27.75	4.94

VFG000165(gb	NP_252915) (pchF) pyochelin synthetase PchF [Pyochelin (VF0095)] [Pseudomonas aeruginosa PAO1]	7.20E-55	2842428	30.89	4.07
VFG001821(gb	NP_216896) (mbtE) Peptide synthetase MbtE (peptide synthase) [Mycobactin (VF0299)] [Mycobacterium tuberculosis H37Rv]	0.00	2843712	28.60	6.27
VFG016041(gb	NP_251092) (pvdI) peptide synthase [pyoverdine (IA001)] [Pseudomonas aeruginosa PAO1]	0.00	2843820	30.80	7.20
VFG016041(gb	NP_251092) (pvdI) peptide synthase [pyoverdine (IA001)] [Pseudomonas aeruginosa PAO1]	0.00	2843823	31.43	8.69
VFG016058(gb	NP_251114) (pvdL) peptide synthase PvdL [pyoverdine (IA001)] [Pseudomonas aeruginosa PAO1]	0.00	2844114	28.72	8.43
VFG001820(gb	NP_216895) (mbtF) Peptide synthetase MbtF (peptide synthase) [Mycobactin (VF0299)] [Mycobacterium tuberculosis H37Rv]	3.80E-59	2844450	26.43	8.44
VFG049160(gb	YP_006635486.1) (clbN) colibactin non-ribosomal peptide synthetase ClbN [Colibactin (VF0573)] [Klebsiella pneumoniae subsp. pneumoniae 1084]	1.90E-73	2844579	22.79	10.79
VFG000930(gb	NP_752604) (entF) enterobactin synthase multienzyme complex component, ATP-dependent [Enterobactin (VF0228)] [Escherichia coli CFT073]	8.94E-110	2844828	27.14	7.42
VFG016038(gb	NP_251090) (pvdJ) pyoverdine biosynthesis protein PvdJ [pyoverdine (IA001)] [Pseudomonas aeruginosa PAO1]	0.00	2845005	32.98	6.15
VFG000161(gb	NP_251089) (pvdD) pyoverdine synthetase D [Pyoverdine (VF0094)] [Pseudomonas aeruginosa PAO1]	0.00	2845029	34.35	4.21
VFG048498(gb	YP_002918370.1) (entF) enterobactin synthase subunit F [Ent (VF0562)] [Klebsiella pneumoniae subsp. pneumoniae NTUH-K2044]	3.92E-108	2845029	27.51	6.26
VFG016058(gb	NP_251114) (pvdL) peptide synthase PvdL [pyoverdine (IA001)] [Pseudomonas aeruginosa PAO1]	1.27E-126	2845041	28.46	7.41
VFG049154(gb	YP_006635480.1) (clbH) colibactin non-ribosomal peptide synthetase ClbH [Colibactin (VF0573)] [Klebsiella pneumoniae subsp. pneumoniae 1084]	6.20E-125	2845077	27.94	4.21
VFG016041(gb	NP_251092) (pvdI) peptide synthase [pyoverdine (IA001)] [Pseudomonas aeruginosa PAO1]	2.99E-119	2845077	29.64	10.00
VFG001403(gb	NP_216899) (mbtB) Phenyloxazoline synthase MbtB (phenyloxazoline synthetase) [Mycobactin (VF0299)] [Mycobacterium tuberculosis H37Rv]	6.50E-67	2845077	28.74	5.44
VFG049156(gb	YP_006635482.1) (clbJ) colibactin non-ribosomal peptide synthetase ClbJ [Colibactin (VF0573)] [Klebsiella pneumoniae subsp. pneumoniae 1084]	0.00	2845080	25.71	7.75
VFG049148(gb	YP_006635474.1) (clbB) colibactin hybrid non-ribosomal peptide synthetase/type I polyketide synthase ClbB [Colibactin (VF0573)] [Klebsiella pneumoniae subsp. pneumoniae 1084]	4.06E-80	2845080	28.21	6.24
VFG049154(gb	YP_006635480.1) (clbH) colibactin non-ribosomal peptide synthetase ClbH [Colibactin (VF0573)] [Klebsiella pneumoniae subsp. pneumoniae 1084]	4.44E-91	2845320	36.94	3.93
VFG049157(gb	YP_006635483.1) (clbK) colibactin hybrid non-ribosomal peptide synthetase/type I polyketide synthase ClbK [Colibactin (VF0573)] [Klebsiella pneumoniae subsp. pneumoniae 1084]	3.64E-77	2845467	24.19	6.86
VFG001821(gb	NP_216896) (mbtE) Peptide synthetase MbtE (peptide synthase) [Mycobactin (VF0299)] [Mycobacterium tuberculosis H37Rv]	0.00	2846901	29.90	5.79
VFG016041(gb	NP_251092) (pvdI) peptide synthase [pyoverdine (IA001)] [Pseudomonas aeruginosa PAO1]	0.00	2846916	30.25	5.86
VFG001820(gb	NP_216895) (mbtF) Peptide synthetase MbtF (peptide synthase) [Mycobactin (VF0299)] [Mycobacterium tuberculosis H37Rv]	5.00E-83	2847258	24.18	9.79
VFG016058(gb	NP_251114) (pvdL) peptide synthase PvdL [pyoverdine (IA001)] [Pseudomonas aeruginosa PAO1]	0.00	2847276	29.28	6.48
VFG016041(gb	NP_251092) (pvdI) peptide synthase [pyoverdine (IA001)] [Pseudomonas aeruginosa PAO1]	0.00	2847582	33.94	5.48
VFG049156(gb	YP_006635482.1) (clbJ) colibactin non-ribosomal peptide synthetase ClbJ [Colibactin (VF0573)] [Klebsiella pneumoniae subsp. pneumoniae 1084]	1.77E-163	2848161	24.96	7.51
VFG016041(gb	NP_251092) (pvdI) peptide synthase [pyoverdine (IA001)] [Pseudomonas aeruginosa PAO1]	8.45E-123	2848161	28.41	9.25
VFG001403(gb	NP_216899) (mbtB) Phenyloxazoline synthase MbtB (phenyloxazoline synthetase) [Mycobactin (VF0299)] [Mycobacterium tuberculosis H37Rv]	4.85E-78	2848164	25.34	10.16
VFG000930(gb	NP_752604) (entF) enterobactin synthase multienzyme complex component, ATP-dependent [Enterobactin (VF0228)] [Escherichia coli CFT073]	4.80E-113	2848170	27.54	6.77
VFG049160(gb	YP_006635486.1) (clbN) colibactin non-ribosomal peptide synthetase ClbN [Colibactin (VF0573)] [Klebsiella pneumoniae subsp. pneumoniae 1084]	1.53E-73	2848176	22.84	8.93
VFG016038(gb	NP_251090) (pvdJ) pyoverdine biosynthesis protein PvdJ [pyoverdine (IA001)] [Pseudomonas aeruginosa PAO1]	0.00	2848182	30.50	6.75
VFG016058(gb	NP_251114) (pvdL) peptide synthase PvdL [pyoverdine (IA001)] [Pseudomonas aeruginosa PAO1]	8.82E-140	2848182	29.95	7.67
VFG049148(gb	YP_006635474.1) (clbB) colibactin hybrid non-ribosomal peptide synthetase/type I polyketide synthase ClbB [Colibactin (VF0573)] [Klebsiella pneumoniae subsp. pneumoniae 1084]	3.41E-79	2848182	27.79	4.09
VFG000161(gb	NP_251089) (pvdD) pyoverdine synthetase D [Pyoverdine (VF0094)] [Pseudomonas aeruginosa PAO1]	0.00	2848185	32.29	5.95
VFG048498(gb	YP_002918370.1) (entF) enterobactin synthase subunit F [Ent (VF0562)] [Klebsiella pneumoniae subsp. pneumoniae NTUH-K2044]	3.50E-109	2848188	27.72	6.55
VFG049154(gb	YP_006635480.1) (clbH) colibactin non-ribosomal peptide synthetase ClbH [Colibactin (VF0573)] [Klebsiella pneumoniae subsp. pneumoniae 1084]	0.00	2848194	29.59	8.07
VFG037442(gb	YP_001847247) (basA) acinetobactin biosynthesis protein [Acinetobactin (VF0467)] [Acinetobacter baumannii ACICU]	1.34E-56	2848230	27.53	7.43
VFG049157(gb	YP_006635483.1) (clbK) colibactin hybrid non-ribosomal peptide synthetase/type I polyketide synthase ClbK [Colibactin (VF0573)] [Klebsiella pneumoniae subsp. pneumoniae 1084]	7.84E-77	2848650	25.55	6.79
VFG000165(gb	NP_252915) (pchF) pyochelin synthetase PchF [Pyochelin (VF0095)] [Pseudomonas aeruginosa PAO1]	4.12E-63	2848659	26.56	9.56
VFG001821(gb	NP_216896) (mbtE) Peptide synthetase MbtE (peptide synthase) [Mycobactin (VF0299)] [Mycobacterium tuberculosis H37Rv]	4.33E-149	2849952	28.60	5.00
VFG016041(gb	NP_251092) (pvdI) peptide synthase [pyoverdine (IA001)] [Pseudomonas aeruginosa PAO1]	3.03E-147	2850345	29.50	6.10
VFG016058(gb	NP_251114) (pvdL) peptide synthase PvdL [pyoverdine (IA001)] [Pseudomonas aeruginosa PAO1]	4.47E-123	2850345	26.22	7.62
VFG001820(gb	NP_216895) (mbtF) Peptide synthetase MbtF (peptide synthase) [Mycobactin (VF0299)] [Mycobacterium tuberculosis H37Rv]	1.98E-64	2850681	24.44	9.74
VFG048498(gb	YP_002918370.1) (entF) enterobactin synthase subunit F [Ent (VF0562)] [Klebsiella pneumoniae subsp. pneumoniae NTUH-K2044]	8.46E-107	2851224	29.52	5.78
VFG049148(gb	YP_006635474.1) (clbB) colibactin hybrid non-ribosomal peptide synthetase/type I polyketide synthase ClbB [Colibactin (VF0573)] [Klebsiella pneumoniae subsp. pneumoniae 1084]	2.90E-68	2851269	26.98	5.92
VFG000930(gb	NP_752604) (entF) enterobactin synthase multienzyme complex component, ATP-dependent [Enterobactin (VF0228)] [Escherichia coli CFT073]	5.42E-104	2851272	28.50	6.08
VFG000161(gb	NP_251089) (pvdD) pyoverdine synthetase D [Pyoverdine (VF0094)] [Pseudomonas aeruginosa PAO1]	1.21E-119	2851278	28.90	4.68
VFG001403(gb	NP_216899) (mbtB) Phenyloxazoline synthase MbtB (phenyloxazoline synthetase) [Mycobactin (VF0299)] [Mycobacterium tuberculosis H37Rv]	5.86E-60	2851281	26.55	6.03
VFG016058(gb	NP_251114) (pvdL) peptide synthase PvdL [pyoverdine (IA001)] [Pseudomonas aeruginosa PAO1]	1.25E-111	2851296	27.22	8.66
VFG016041(gb	NP_251092) (pvdI) peptide synthase [pyoverdine (IA001)] [Pseudomonas aeruginosa PAO1]	6.49E-107	2851338	28.57	4.83
VFG049154(gb	YP_006635480.1) (clbH) colibactin non-ribosomal peptide synthetase ClbH [Colibactin (VF0573)] [Klebsiella pneumoniae subsp. pneumoniae 1084]	2.62E-80	2851350	25.03	5.93
VFG049160(gb	YP_006635486.1) (clbN) colibactin non-ribosomal peptide synthetase ClbN [Colibactin (VF0573)] [Klebsiella pneumoniae subsp. pneumoniae 1084]	1.11E-74	2851377	23.26	6.77

VFG037442(gb	YP_001847247) (basA) acinetobactin biosynthesis protein [Acinetobactin (VF0467)] [Acinetobacter baumannii ACICU]	7.44E-56	2851542	27.94	7.32
VFG049157(gb	YP_006635483.1) (clbK) colibactin hybrid non-ribosomal peptide synthetase/type I polyketide synthase ClbK [Colibactin (VF0573)] [Klebsiella pneumoniae subsp. pneumoniae 1084]	9.14E-57	2851734	31.24	2.10
VFG047256(gb	YP_169432.1) (glmU) UDP-N-acetylglucosamine pyrophosphorylase/glucosamine-1-phosphate N-acetyltransferase [LPS (VF0542)] [Francisella tularensis subsp. tularensis SCHU S4]	2.38E-102	2924680	39.86	1.35

Supplemental Table S2: VFDB screening at amino acid level for *Limosilactobacillus fermentum* LMG6902

Hit	Description	E-value	Query start	%Identity	%Gaps
VFG001859(gb	YP_096662) (feoB) ferrous iron transporter B [FeoAB (VF0160)] [Legionella pneumophila subsp. pneumophila str. Philadelphia 1]	8.63E-100	6908	33.24	10.71
VFG047401(gb	YP_169301.1) (feoB) Fe(2+) transporter permease subunit FeoB [FupA (VF0550)] [Francisella tularensis subsp. tularensis SCHU S4]	1.97E-103	6920	34.13	10.86
VFG002158(gb	NP_464456) (lplA1) lipoate protein ligase [LplA1 (VF0347)] [Listeria monocytogenes EGD-e]	3.44E-91	48988	47.24	1.84
VFG005197(gb	NP_358462) (pavA) Fibronectin-binding protein-like protein A [PavA (VF0283)] [Streptococcus pneumoniae R6]	5.63E-135	65500	45.15	3.88
VFG002160(gb	NP_465354) (fbpA) fibronectin-binding protein [FbpA (VF0349)] [Listeria monocytogenes EGD-e]	5.70E-129	65521	43.19	2.12
VFG005177(gb	NP_664456) (fbp54) fibronectin-bing protein FbaA [Fibronectin-binding proteins (CVF113)] [Streptococcus pyogenes MGAS315]	3.25E-128	65521	43.29	6.18
VFG000959(gb	NP_269190) (fbp54) fibronectin-bing protein Fbp54 [FBPs (VF0243)] [Streptococcus pyogenes M1 GAS]	4.26E-128	65521	43.29	6.18
VFG047708(gb	YP_170571.1) (carB) carbamoyl phosphate synthase large subunit [Pyrimidine biosynthesis (VF0558)] [Francisella tularensis subsp. tularensis SCHU S4]	7.63E-71	72492	26.35	4.78
VFG047726(gb	YP_170570.1) (carA) carbamoyl phosphate synthase small subunit [Pyrimidine biosynthesis (VF0558)] [Francisella tularensis subsp. tularensis SCHU S4]	8.99E-61	74380	35.84	11.17
VFG015551(gb	NP_248741) (phzH) phenazine-modifying enzyme [Phenazines biosynthesis (CVF536)] [Pseudomonas aeruginosa PAO1]	7.02E-54	96730	27.90	13.57
VFG047111(gb	YP_170393.1) (wbtH) asparagine synthase (glutamine-hydrolyzing) [LPS (VF0542)] [Francisella tularensis subsp. tularensis SCHU S4]	1.10E-76	96742	29.89	9.40
VFG000080(gb	NP_464522) (clpE) ATP-dependent protease [ClpE (VF0073)] [Listeria monocytogenes EGD-e]	0.00	144070	48.13	6.32
VFG038395(gb	YP_856375) (clpB) type VI secretion system ATPase ClpV1 [T6SS (SS194)] [Aeromonas hydrophila subsp. hydrophila ATCC 7966]	1.75E-57	144163	42.96	2.46
VFG000079(gb	NP_463763) (clpC) endopeptidase Clp ATP-binding chain C [ClpC (VF0072)] [Listeria monocytogenes EGD-e]	2.12E-165	144190	45.97	5.94
VFG002084(gb	NP_232517) (clpB/vasG) type VI secretion system AAA+ family ATPase [T6SS (VF0335)] [Vibrio cholerae O1 biovar El Tor str. N16961]	1.64E-61	144223	44.44	1.53
VFG048693(gb	YP_005226603.1) (clpV/tssH) type VI secretion system ATPase TssH [T6SS (VF0569)] [Klebsiella pneumoniae subsp. pneumoniae HS11286]	6.52E-58	144253	45.20	1.60
VFG049904(gb	YP_309261.1) (clpV/tssH) Type VI secretion system ATPase ClpV/TssH [T6SS (VF0579)] [Shigella sonnei Ss046]	9.69E-61	144262	47.98	1.61
VFG002480(gb	YP_111509) (tssH-5/clpV) Clp-type ATPase chaperone protein [T6SS-1 (VF0429)] [Burkholderia pseudomallei K96243]	4.41E-54	144265	45.93	1.63
VFG002076(gb	NP_248780) (clpV1) type VI secretion system AAA+ family ATPase [HSI-I (VF0334)] [Pseudomonas aeruginosa PAO1]	3.35E-55	144271	45.02	1.99
VFG012509(gb	NP_753167) (iroC) ATP binding cassette transporter [Salmochelin (IA013)] [Escherichia coli CFT073]	2.96E-64	206169	29.70	1.39
VFG013248(gb	NP_438233) (msbA) lipid transporter ATP-binding/permease [LOS (CVF494)] [Haemophilus influenzae Rd KW20]	1.36E-79	206178	32.04	1.17
VFG000366(gb	NP_405475) (ybtQ) yersiniabactin ABC transporter ATP-binding/permease protein YbtQ [Yersiniabactin (VF0136)] [Yersinia pestis CO92]	1.31E-58	206178	30.52	5.82
VFG044325(gb	YP_002920245) (ybtQ) yersiniabactin ABC transporter ATP-binding/permease protein YbtQ [Ybt (VF0564)] [Klebsiella pneumoniae subsp. pneumoniae NTUH-K2044]	1.52E-58	206178	30.52	5.82
VFG001269(gb	NP_879579) (cyaB) cyclolysin secretion ATP-binding protein [Cya (VF0028)] [Bordetella pertussis Tohama I]	2.26E-73	206187	31.26	1.36
VFG007023(gb	NP_231091) (rtxB) RTX toxin transporter RtxB [RTX toxin (CVF263)] [Vibrio cholerae O1 biovar El Tor str. N16961]	2.21E-64	206187	29.70	2.07
VFG038918(gb	YP_855893) (rtxE) RTX toxin transporter, ATPase protein [The repeat in toxin (RTX) (CVF795)] [Aeromonas hydrophila subsp. hydrophila ATCC 7966]	4.61E-63	206187	28.68	2.10
VFG038916(gb	YP_855895) (rtxB) RTX toxin transporter, ATPase protein [The repeat in toxin (RTX) (CVF795)] [Aeromonas hydrophila subsp. hydrophila ATCC 7966]	9.22E-63	206187	31.71	2.44
VFG000841(gb	YP_325609) (hlyB) hemolysin transport protein [Hemolysin (VF0207)] [Escherichia coli O157:H7 str. EDL933]	1.27E-62	206187	31.12	2.81
VFG000907(gb	NP_755448) (hlyB) Hemolysin B [<alpha>-Hemolysin (VF0225)] [Escherichia coli CFT073]	3.62E-62	206187	28.60	1.15
VFG009570(gb	NP_215864) (irtA) Iron-regulated transporter IrtA [mycobactin (IA031)] [Mycobacterium tuberculosis H37Rv]	5.18E-55	206190	32.71	4.67
VFG044336(gb	YP_002920268) (iroC) ABC transporter [Sal (VF0563)] [Klebsiella pneumoniae subsp. pneumoniae NTUH-K2044]	7.50E-63	206193	29.56	1.34
VFG000365(gb	NP_405474) (ybtP) yersiniabactin ABC transporter ATP-binding/permease protein YbtP [Yersiniabactin (VF0136)] [Yersinia pestis CO92]	1.05E-54	206193	29.18	5.45
VFG044326(gb	YP_002920246) (ybtP) yersiniabactin ABC transporter ATP-binding/permease protein YbtP [Ybt (VF0564)] [Klebsiella pneumoniae subsp. pneumoniae NTUH-K2044]	3.87E-54	206193	28.99	5.45
VFG044336(gb	YP_002920268) (iroC) ABC transporter [Sal (VF0563)] [Klebsiella pneumoniae subsp. pneumoniae NTUH-K2044]	1.21E-51	206220	28.37	3.82
VFG002190(gb	NP_816141) (cpsA) undecaprenyl diphosphate synthase [Capsule (VF0361)] [Enterococcus faecalis V583]	9.55E-96	247402	59.60	0.80
VFG002189(gb	NP_816140) (cpsB) phosphatidate cytidylyltransferase [Capsule (VF0361)] [Enterococcus faecalis V583]	1.10E-75	248174	51.67	3.72
VFG047726(gb	YP_170570.1) (carA) carbamoyl phosphate synthase small subunit [Pyrimidine biosynthesis (VF0558)] [Francisella tularensis subsp. tularensis SCHU S4]	4.31E-67	261038	37.80	11.02
VFG047708(gb	YP_170571.1) (carB) carbamoyl phosphate synthase large subunit [Pyrimidine biosynthesis (VF0558)] [Francisella tularensis subsp. tularensis SCHU S4]	0.00	262098	46.48	3.00
VFG037118(gb	NP_273782) (recN) DNA repair protein RecN [RecN (VF0457)] [Neisseria meningitidis MC58]	6.75E-66	316915	30.81	3.35
VFG047256(gb	YP_169432.1) (glmU) UDP-N-acetylglucosamine pyrophosphorylase/glucosamine-1-phosphate N-acetyltransferase [LPS (VF0542)] [Francisella tularensis subsp. tularensis SCHU S4]	2.89E-98	463402	40.91	1.82
VFG001826(gb	NP_217099) (relA) Probable GTP pyrophosphokinase RelA (ATP:GTP 3'-pyrophosphotransferase) (PPGPP synthetase I) ((P)PPGPP synthetase) (GTP diphosphokinase) [RelA (VF0287)] [Mycobacterium tuberculosis H37Rv]	0.00	490617	40.19	3.18
VFG002158(gb	NP_464456) (lplA1) lipoate protein ligase [LplA1 (VF0347)] [Listeria monocytogenes EGD-e]	3.42E-71	512248	38.17	2.37
VFG000670(gb	NP_706258) (gtrB) bactoprenol glucosyl transferase [LPS (VF0124)] [Shigella flexneri 2a str. 301]	4.83E-53	636047	41.72	1.32
VFG000670(gb	NP_706258) (gtrB) bactoprenol glucosyl transferase [LPS (VF0124)] [Shigella flexneri 2a str. 301]	8.21E-52	638183	36.33	5.14
VFG013368(gb	NP_439034) (rffG) dTDP-glucose 46-dehydratase [LOS (CVF494)] [Haemophilus influenzae Rd KW20]	2.42E-79	644762	44.38	7.29
VFG047022(gb	YP_170387.1) (wbtM) dTDP-D-glucose 4,6-dehydratase [LPS (VF0542)] [Francisella tularensis subsp. tularensis SCHU S4]	2.94E-81	644765	43.21	5.86

VFG000080(gb	NP_464522) (clpE) ATP-dependent protease [ClpE (VF0073)] [Listeria monocytogenes EGD-e]	0.00	656234	57.46	2.07
VFG048693(gb	YP_005226603.1) (clpV/tssH) type VI secretion system ATPase TssH [T6SS (VF0569)] [Klebsiella pneumoniae subsp. pneumoniae HS11286]	1.38E-124	656270	39.63	8.08
VFG000079(gb	NP_463763) (clpC) endopeptidase Clp ATP-binding chain C [ClpC (VF0072)] [Listeria monocytogenes EGD-e]	0.00	656276	51.14	2.28
VFG038395(gb	YP_856375) (clpB) type VI secretion system ATPase ClpV1 [T6SS (SS194)] [Aeromonas hydrophila subsp. hydrophila ATCC 7966]	2.69E-112	656315	36.50	11.76
VFG002084(gb	NP_232517) (clpB/vasG) type VI secretion system AAA+ family ATPase [T6SS (VF0335)] [Vibrio cholerae O1 biovar El Tor str. N16961]	3.08E-113	656453	38.78	10.89
VFG002076(gb	NP_248780) (clpV1) type VI secretion system AAA+ family ATPase [HSI-I (VF0334)] [Pseudomonas aeruginosa PAO1]	3.61E-61	657152	43.44	5.62
VFG002480(gb	YP_111509) (tssH-5/clpV) Clp-type ATPase chaperone protein [T6SS-1 (VF0429)] [Burkholderia pseudomallei K96243]	2.02E-64	657251	51.26	4.33
VFG049904(gb	YP_309261.1) (clpV/tssH) Type VI secretion system ATPase ClpV/TssH [T6SS (VF0579)] [Shigella sonnei Ss046]	9.64E-70	657299	51.14	1.89
VFG046465(gb	YP_169203.1) (tufA) elongation factor Tu [EF-Tu (VF0460)] [Francisella tularensis subsp. tularensis SCHU S4]	5.80E-172	668961	68.45	0.25
VFG006717(gb	NP_465159) (lap) Listeria adhesion protein Lap [Lap (VF0444)] [Listeria monocytogenes EGD-e]	0.00	841874	58.58	0.35
VFG002361(gb	YP_001007253) (galE) UDP-glucose 4-epimerase [O-antigen (VF0392)] [Yersinia enterocolitica subsp. enterocolitica 8081]	6.32E-78	862888	44.41	5.00
VFG013286(gb	NP_438515) (galE) UDP-glucose 4-epimerase [LOS (CVF494)] [Haemophilus influenzae Rd KW20]	5.69E-71	862888	42.23	4.40
VFG000314(gb	NP_207158) (gluE) UDP-glucose 4-epimerase [LPS (VF0056)] [Helicobacter pylori 26695]	1.93E-60	862888	36.87	5.60
VFG046645(gb	YP_169798.1) (galE) UDP-glucose 4-epimerase GalE [Capsule (VF0543)] [Francisella tularensis subsp. tularensis SCHU S4]	1.07E-69	862894	42.86	3.34
VFG002158(gb	NP_464456) (lplA1) lipoate protein ligase [LplA1 (VF0347)] [Listeria monocytogenes EGD-e]	1.80E-95	910028	49.54	1.23
VFG039536(gb	NP_820549) (CBU_1566) Coxiella Dot/Icm type IVB secretion system translocated effector [T4SS effectors (CVF803)] [Coxiella burnetii RSA 493]	1.75E-51	937945	43.51	1.26
VFG001321(gb	YP_001332078) (isdE) iron-regulated surface determinant protein E [Isd (VF0015)] [Staphylococcus aureus subsp. aureus str. Newman]	1.45E-66	1003747	44.57	1.16
VFG048488(gb	YP_002918371.1) (fepC) iron-enterobactin transporter ATP-binding protein [Ent (VF0562)] [Klebsiella pneumoniae subsp. pneumoniae NTUH-K2044]	1.32E-51	1005426	34.80	0.80
VFG000925(gb	NP_752606) (fepC) ferrienterobactin ABC transporter ATPase [Enterobactin (VF0228)] [Escherichia coli CFT073]	6.18E-51	1005435	35.63	0.81
VFG047726(gb	YP_170570.1) (carA) carbamoyl phosphate synthase small subunit [Pyrimidine biosynthesis (VF0558)] [Francisella tularensis subsp. tularensis SCHU S4]	4.76E-77	1037292	39.52	6.37
VFG047708(gb	YP_170571.1) (carB) carbamoyl phosphate synthase large subunit [Pyrimidine biosynthesis (VF0558)] [Francisella tularensis subsp. tularensis SCHU S4]	0.00	1038362	47.73	3.89
VFG000077(gb	NP_465991) (clpP) ATP-dependent Clp protease proteolytic subunit [ClpP (VF0074)] [Listeria monocytogenes EGD-e]	7.50E-79	1085339	67.89	0.00
VFG013327(gb	NP_438900) (yhxB/manB) phosphomannomutase [LOS (CVF494)] [Haemophilus influenzae Rd KW20]	1.11E-75	1097132	32.64	10.88
VFG000964(gb	NP_270109) (hasC) UDP-glucose pyrophosphorylase [Hyaluronic acid capsule (VF0244)] [Streptococcus pyogenes M1 GAS]	9.44E-136	1108902	71.48	0.34
VFG013346(gb	NP_438972) (galU) glucosephosphate uridylyltransferase [LOS (CVF494)] [Haemophilus influenzae Rd KW20]	1.79E-68	1108902	43.84	2.74
VFG048990(gb	YP_002920369.1) (galF) UTP-glucose-1-phosphate uridylyltransferase subunit GalF [Capsule (VF0560)] [Klebsiella pneumoniae subsp. pneumoniae NTUH-K2044]	6.44E-62	1108905	43.34	4.10
VFG001855(gb	YP_094724) (htpB) Hsp60, 60K heat shock protein HtpB [Hsp60 (VF0159)] [Legionella pneumophila subsp. pneumophila str. Philadelphia 1]	1.63E-166	1123212	55.98	0.39
VFG038840(gb	YP_008043465) (flmH) flagellar-related 3-oxoacyl-ACP reductase [Polar flagella (VF0473)] [Aeromonas hydrophila ML09-119]	7.47E-54	1135800	43.85	2.46
VFG048830(gb	YP_002920353.1) (gnd) 6-phosphogluconate dehydrogenase [Capsule (VF0560)] [Klebsiella pneumoniae subsp. pneumoniae NTUH-K2044]	0.00	1303310	64.33	1.49
VFG032992(gb	NP_464816) (oatA) peptidoglycan O-acetyltransferase [OatA (VF0441)] [Listeria monocytogenes EGD-e]	2.34E-72	1410997	32.75	3.67
VFG000079(gb	NP_463763) (clpC) endopeptidase Clp ATP-binding chain C [ClpC (VF0072)] [Listeria monocytogenes EGD-e]	0.00	1459539	55.09	1.84
VFG000080(gb	NP_464522) (clpE) ATP-dependent protease [ClpE (VF0073)] [Listeria monocytogenes EGD-e]	4.55E-180	1460046	49.76	2.57
VFG048693(gb	YP_005226603.1) (clpV/tssH) type VI secretion system ATPase TssH [T6SS (VF0569)] [Klebsiella pneumoniae subsp. pneumoniae HS11286]	5.39E-140	1460046	43.24	8.09
VFG002076(gb	NP_248780) (clpV1) type VI secretion system AAA+ family ATPase [HSI-I (VF0334)] [Pseudomonas aeruginosa PAO1]	2.75E-129	1460046	39.60	11.88
VFG038395(gb	YP_856375) (clpB) type VI secretion system ATPase ClpV1 [T6SS (SS194)] [Aeromonas hydrophila subsp. hydrophila ATCC 7966]	4.01E-118	1460046	38.94	11.06
VFG002084(gb	NP_232517) (clpB/vasG) type VI secretion system AAA+ family ATPase [T6SS (VF0335)] [Vibrio cholerae O1 biovar El Tor str. N16961]	2.81E-115	1460046	37.93	11.51
VFG049904(gb	YP_309261.1) (clpV/tssH) Type VI secretion system ATPase ClpV/TssH [T6SS (VF0579)] [Shigella sonnei Ss046]	4.73E-62	1460046	51.56	0.39
VFG002480(gb	YP_111509) (tssH-5/clpV) Clp-type ATPase chaperone protein [T6SS-1 (VF0429)] [Burkholderia pseudomallei K96243]	5.67E-64	1460052	55.28	0.41
VFG002480(gb	YP_111509) (tssH-5/clpV) Clp-type ATPase chaperone protein [T6SS-1 (VF0429)] [Burkholderia pseudomallei K96243]	3.39E-67	1460922	40.92	4.32
VFG000574(gb	NP_462662) (mgtB) Mg2+ transport protein [MgtBC (VF0106)] [Salmonella enterica subsp. enterica serovar Typhimurium str. LT2]	8.39E-61	1505986	27.33	11.43
VFG049062(gb	YP_002920345.1) (glf) UDP-galactopyranose mutase [LPS (VF0561)] [Klebsiella pneumoniae subsp. pneumoniae NTUH-K2044]	2.48E-82	1665225	40.43	2.66
VFG002182(gb	NP_816133) (cpsI) UDP-galactopyranose mutase [Capsule (VF0361)] [Enterococcus faecalis V583]	1.24E-160	1665231	64.27	0.80
VFG001967(gb	NP_282580) (glf) UDP-galactopyranose mutase [Capsule (VF0323)] [Campylobacter jejuni subsp. jejuni NCTC 11168]	4.71E-140	1665231	56.79	1.63
VFG047564(gb	YP_169890.1) (purM) phosphoribosylaminoimidazole synthetase [Purine biosynthesis (VF0559)] [Francisella tularensis subsp. tularensis SCHU S4]	6.22E-63	1713721	35.71	1.19

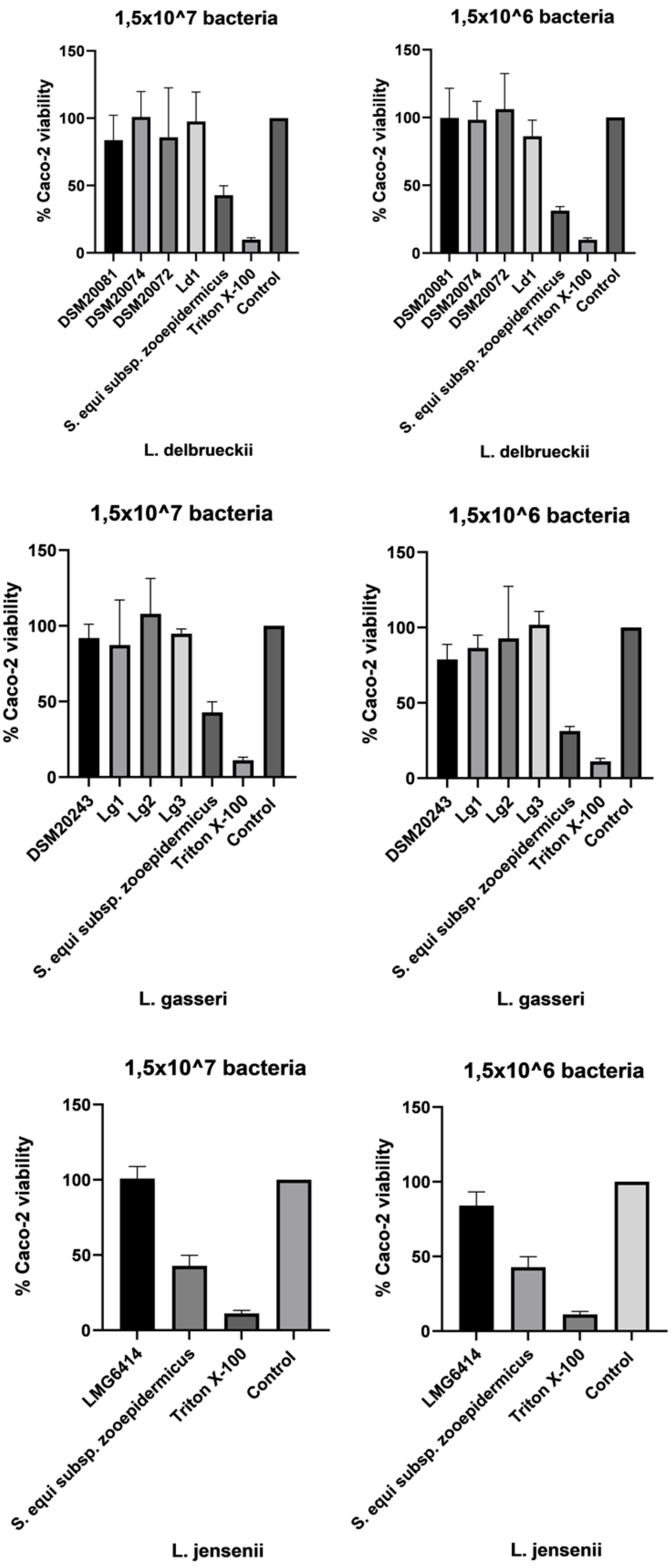
1,5x10^7 bacteria
% Caco-2 viability
150
100
50
0
DSM20081
DSM20074
DSM20072
Ld1
S. equi subsp. zooepidermicus
Triton X-100
Control
L. delbrueckii
1,5x10^6 bacteria
% Caco-2 viability
150
100
50
0
DSM20081
DSM20074
DSM20072
Ld1
S. equi subsp. zooepidermicus
Triton X-100
Control
L. delbrueckii
1,5x10^7 bacteria
% Caco-2 viability
150
100
50
0
DSM20243
Lg1
Lg2
Lg3
S. equi subsp. zooepidermicus
Triton X-100
Control
L. gasseri
1,5x10^6 bacteria
% Caco-2 viability
150
100
50
0
DSM20243
Lg1
Lg2
Lg3
S. equi subsp. zooepidermicus
Triton X-100
Control
L. gasseri
1,5x10^7 bacteria
% Caco-2 viability
150
100
50
0
LMG6414
S. equi subsp. zooepidermicus
Triton X-100
Control
L. jensenii
1,5x10^6 bacteria
% Caco-2 viability
150
100
50
0
LMG6414
S. equi subsp. zooepidermicus
Triton X-100
Control
L. jensenii

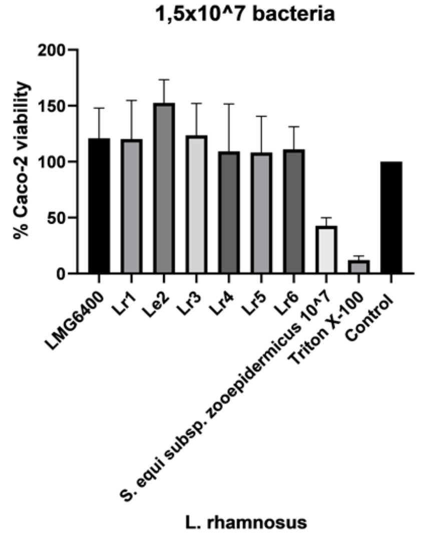
1,5x10^7 bacteria
% Caco-2 viability
0
50
100
150
200
LMG6400
Lr1
Le2
Lr3
Lr4
Lr5
Lr6
S. equi subsp. zooepidermicus 10^7
Triton X-100
Control
L. rhamnosus

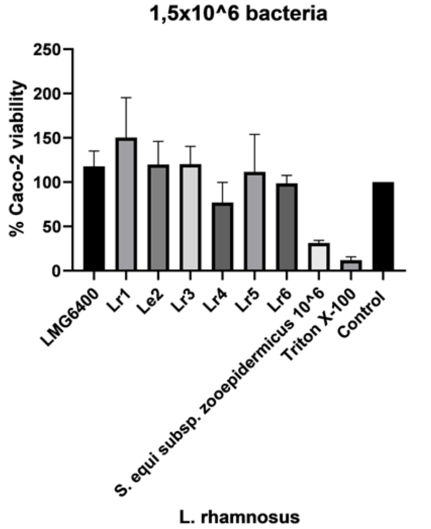
1,5x10^6 bacteria
% Caco-2 viability
0
50
100
150
200
250
LMG6400
Lr1
Le2
Lr3
Lr4
Lr5
Lr6
S. equi subsp. zooepidermicus 10^6
Triton X-100
Control
L. rhamnosus

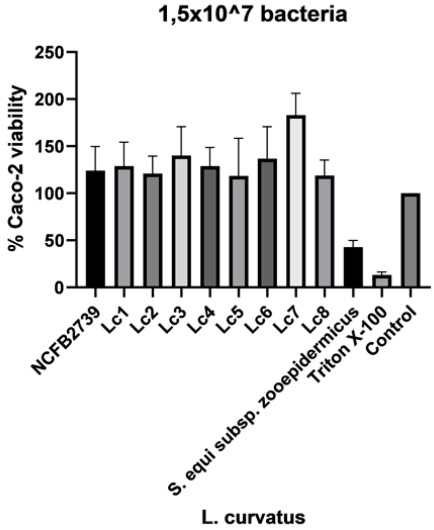
1,5x10^7 bacteria
% Caco-2 viability
0
50
100
150
200
250
NCFB2739
Lc1
Lc2
Lc3
Lc4
Lc5
Lc6
Lc7
Lc8
S. equi subsp. zooepidermicus
Triton X-100
Control
L. curvatus

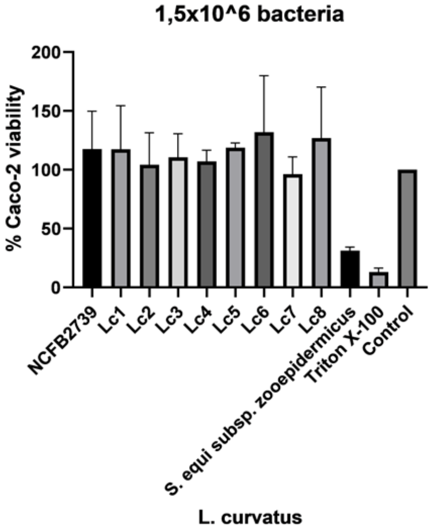
1,5x10^6 bacteria
% Caco-2 viability
0
50
100
150
200
NCFB2739
Lc1
Lc2
Lc3
Lc4
Lc5
Lc6
Lc7
Lc8
S. equi subsp. zooepidermicus
Triton X-100
Control
L. curvatus

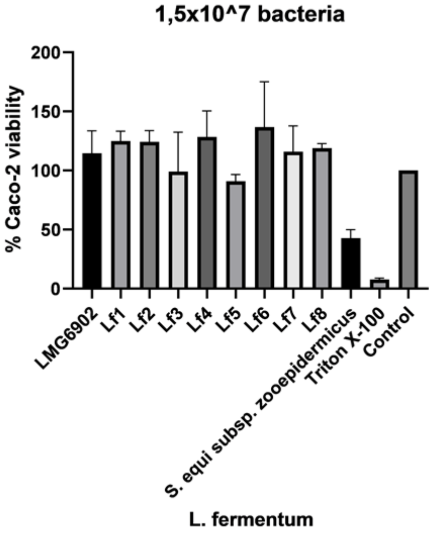
1,5x10^7 bacteria
% Caco-2 viability
0
50
100
150
200
LMG6902
Lf1
Lf2
Lf3
Lf4
Lf5
Lf6
Lf7
Lf8
S. equi subsp. zooepidermicus
Triton X-100
Control
L. fermentum

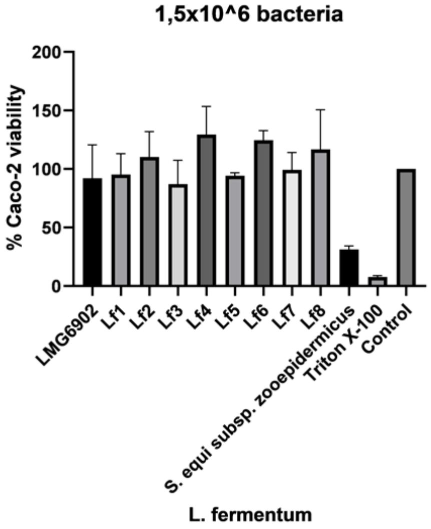
1,5x10^6 bacteria
% Caco-2 viability
0
50
100
150
200
LMG6902
Lf1
Lf2
Lf3
Lf4
Lf5
Lf6
Lf7
Lf8
S. equi subsp. zooepidermicus
Triton X-100
Control
L. fermentum

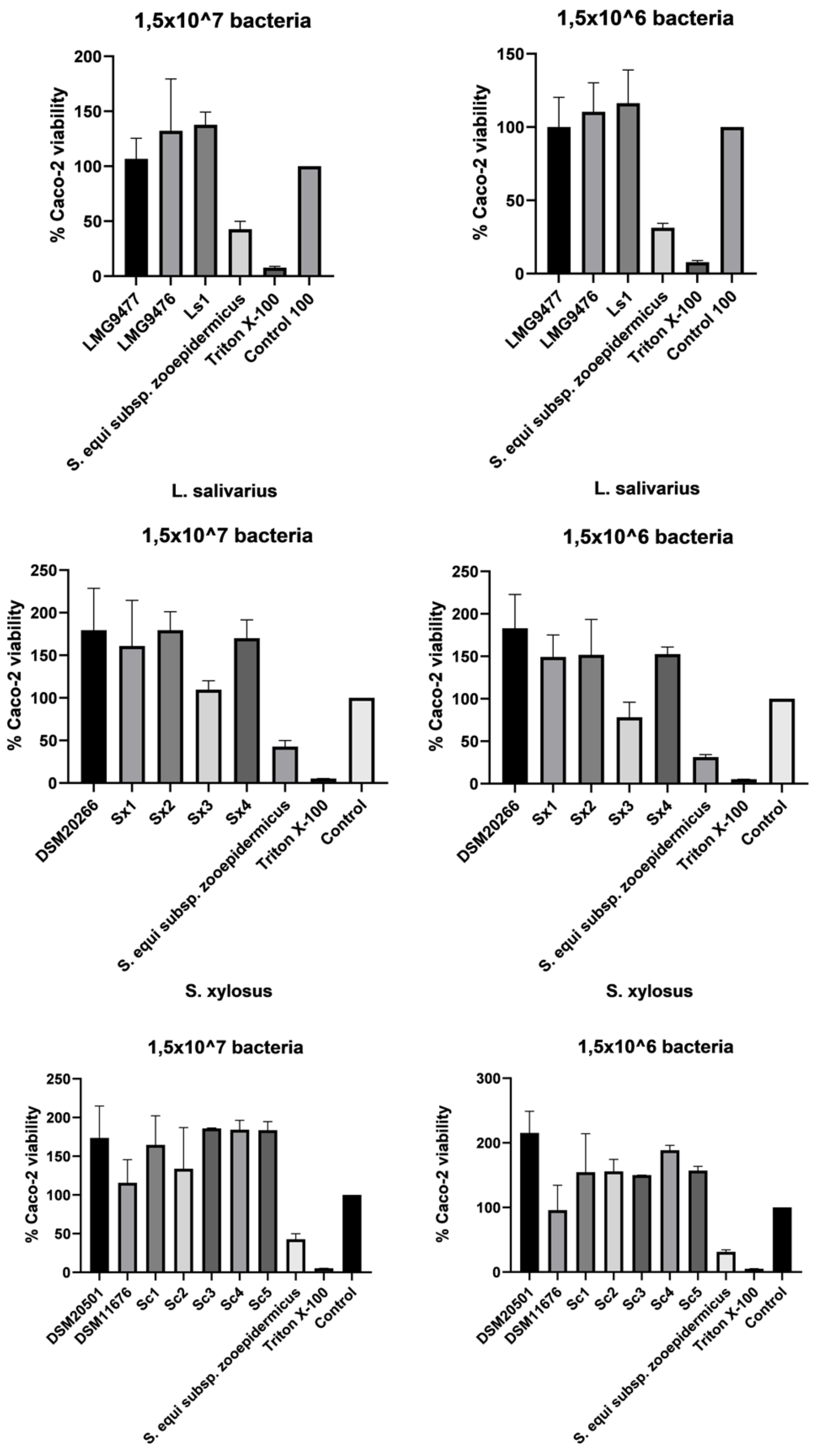

Supplemental Figure S1: Caco-2 cell viability (%) measured through the MTT assay after exposure to the examined strains. *Streptococcus equi* subsp. *zooepidermicus* was included as a pathogenic control, and Triton X-100 treated cells was included as a lysis control, while the control was untreated Caco-2 cells.
